# Hit Identification of a Novel Quinazoline Sulfonamide as a Promising EphB3 Inhibitor: Design, Virtual Combinatorial Library, Synthesis, Biological Evaluation, and Docking Simulation Studies

**DOI:** 10.3390/ph14121247

**Published:** 2021-11-30

**Authors:** Kyeong Lee, Hossam Nada, Hyun Jung Byun, Chang Hoon Lee, Ahmed Elkamhawy

**Affiliations:** 1 BK21 FOUR Team and Integrated Research Institute for Drug Development, College of Pharmacy, Dongguk University-Seoul, Goyang 10326, Korea; kaylee@dongguk.edu (K.L.); bhj1052@dongguk.edu (H.J.B.); uatheone@dongguk.edu (C.H.L.); 2 College of Pharmacy, Dongguk University-Seoul, Goyang 10326, Korea; hossam_hammouda@dgu.ac.kr; 3 Department of Pharmaceutical Organic Chemistry, Faculty of Pharmacy, Mansoura University, Mansoura 35516, Egypt

**Keywords:** EphB3, kinase inhibitors, quinazoline sulfonamide, MM-GBSA calculations, selectivity profile, virtual combinatorial library

## Abstract

EphB3 is a major key player in a variety of cellular activities, including cell migration, proliferation, and apoptosis. However, the exact role of EphB3 in cancer remains ambiguous. Accordingly, new EphB3 inhibitors can increase the understanding of the exact roles of the receptor and may act as promising therapeutic candidates. Herein, a hybrid approach of structure-based design and virtual combinatorial library generated 34 quinazoline sulfonamides as potential selective EphB3 inhibitors. A molecular docking study over EphB3 predicted the binding affinities of the generated library, and the top seven hit compounds (**3a** and **4a**–**f**), with GlideScore ≥ −6.20 Kcal/mol, were chosen for further MM-GBSA calculations. Out of the seven top hits, compound **4c** showed the highest MM-GBSA binding free energy (−74.13 Kcal/mol). To validate these predicted results, compounds **3a** and **4a**–**f** were synthesized and characterized using NMR, HRMS, and HPLC. The biological evaluation revealed compound **4c** as a potent EphB3 inhibitory lead (IC_50_ = 1.04 µM). The screening of **4c** over a mini-panel of kinases consisting of EGFR, Aurora A, Aurora B, CDK2/cyclin A, EphB1, EphB2, EphB4, ERBB2/HER2, and KDR/VEGFR2, showed a promising selective profile against EphB3 isoform. A dose-dependent assay of compound **4c** and a molecular docking study over the different forms of EphB provided insights into the elicited biological activities and highlighted reasonable explanations of the selectivity.

## 1. Introduction

**Figure 1:**
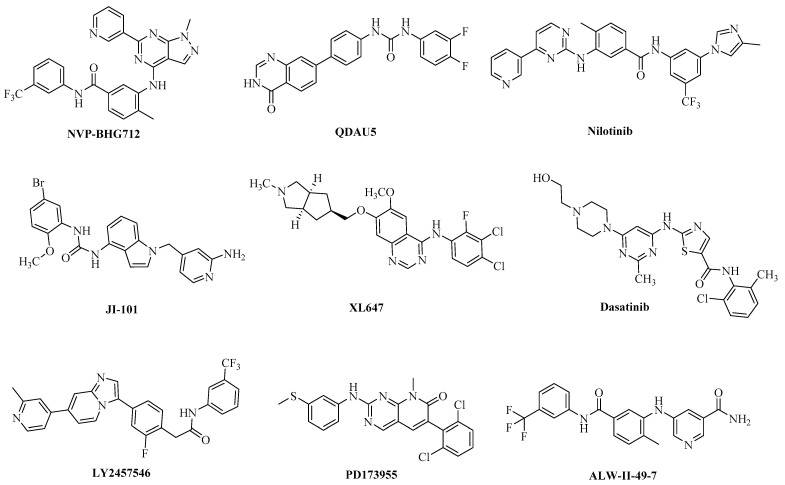
Reported small molecules as Eph inhibitors [18,19,20,21,22,23,24,25,26].

Erythropoietin-producing hepatocellular carcinoma type-B receptor 3 (EphB3) belongs to the Eph receptor tyrosine kinase family, which is divided into classes A and B according to their sequence homology and ligand-binding affinity [1,2]. EphB3 and its ligands were found to be key players in a variety of cellular activities including cell migration, proliferation, and apoptosis. The EphB3 receptor subtype is expressed during embryonic development and in discrete areas of the adult brain, including the cerebellum and hippocampus. It co-localizes to brain regions with high levels of ephrin B ligand expression [3,4]. Several EphB3-related physiological and pathological processes are involved with the development and disease of many organ systems. Overexpression of EphB3 in non-small-cell lung cancer was reported to increase metastasis via increasing the tumor migratory capabilities, which in turn increases the tumor survival [5]. A recent study by Lee et al. reported EphB3 upregulation in gastric cancer with acquired resistance to AZD4547 (a selective inhibitor of a fibroblast growth factor receptor) [6]. In the colorectal cancer mice model, EphB3 expression was also found to be increased along with tumor development [7] and coamplified with PIK3CA in head and neck squamous cell carcinoma (HNSCC) [8]. In addition, Zhao et al. recently reported a potential function of EphB3 in carcinogenesis of ovarian serous carcinoma [9]. Li et al. also reported EphB3 as a tumor promoter in papillary thyroid cancer (PTC) via enhancing the in vitro migration and the in vivo metastasis of PTC cells through regulating the activities of Vav2 and Rho GTPases in a kinase-dependent manner [10]. Moreover, silencing of EphB3 significantly affected cycle progression distribution and increased apoptosis in CRC cell which revealed that knockdown of EphB3 inhibited tumor growth [11,12]. Despite these finding, the exact role of EphB3 in cancer remains controversial since some reports have documented conflicting roles of EphB3 in carcinogenesis [13,14,15,16,17]. Accordingly, EphB3 inhibitors can help to understand the exact roles of the receptor and may act as promising therapeutic candidates. Even though numerous Eph kinase inhibitors have been described in the literature (Figure 1) [18,19,20,21,22,23,24,25,26], almost all suffer from a lack of selectivity for the different Eph isoforms and/or other kinases due to the great conservation within the kinase domains. Unfortunately, the absence of a well-diversified library of isoform-selective inhibitors has hampered the pharmacological mapping of individual Eph kinase activities [27].

**Figure 2:**
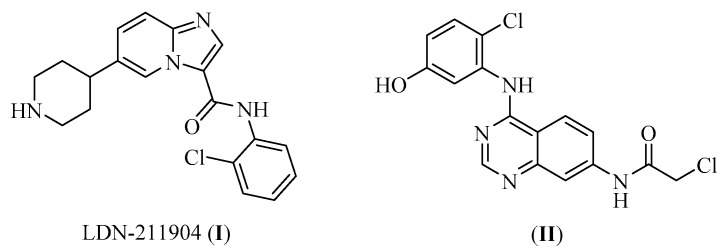
Reported small molecules as selective EphB3 inhibitors.

The investigation of the exact role of EphB3 isoforms has been hampered by the unavailability of many identified selective EphB3 inhibitors. Searching the literature, only two studies successfully reported small molecules as selective EphB3 inhibitors (Figure 2) [15,28]. Accordingly, identification of new small molecules able to reversibly inhibit EphB3 would be a step toward the development of new selective inhibitory candidates. This in turn would further enhance the understanding of the exact functions of the receptor and offer potential therapeutic agents for several cancer disorders related to EphB3.

**Figure 3:**
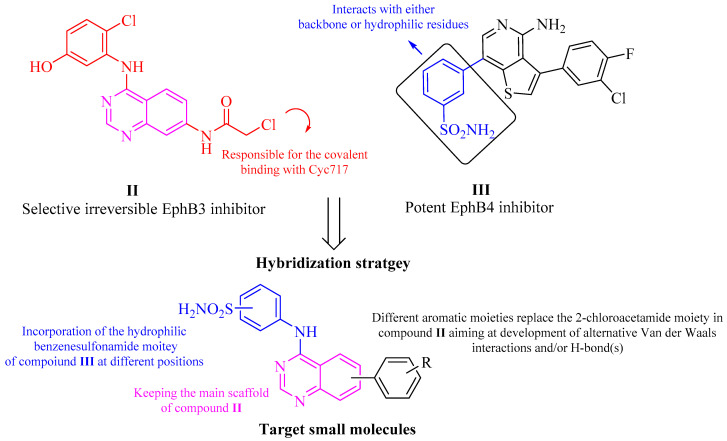
Design of the target small molecules via applying a structural hybridization strategy of compounds **II** and **III**.

Consequently, our aim in this study was to design and synthesize new small molecules as potential EphB3 selective inhibitors with a reversible mode of action. Thus, the quinazoline ring of compound **II** was used as a main scaffold of our target compounds since compound **II** showed a significant selectivity among Eph subtypes. The 2-chloroacetamide moiety in compound **II**, which is responsible for the covalent binding with Cys717 in EphB3 was replaced with several aromatic moieties aiming at development of alternative hydrophobic interactions (Van der Waals interactions) and/or hydrogen bond(s). Moreover, since the phenyl sulfonamide moiety was found in compound **III** (a potent EphB4 inhibitor [29], Figure 3) to interact with either backbone or hydrophilic residues in the binding pocket to improve the ligand-solubility properties, a structural hybridization-based strategy was applied via incorporating the phenyl sulfonamide moiety of compound **III** at position 4 of compound **II** (Figure 3). Herein, we report a new series of small molecules was designed, synthesized, and biologically evaluated as potential selective and reversible EphB3 inhibitors.

## 2. Results and Discussion

### 2.1. Validation of the Crystal Structures and Generation of a Virtual Library

#### 2.1.1. Validation of the Protein Structure

**Figure 4:**
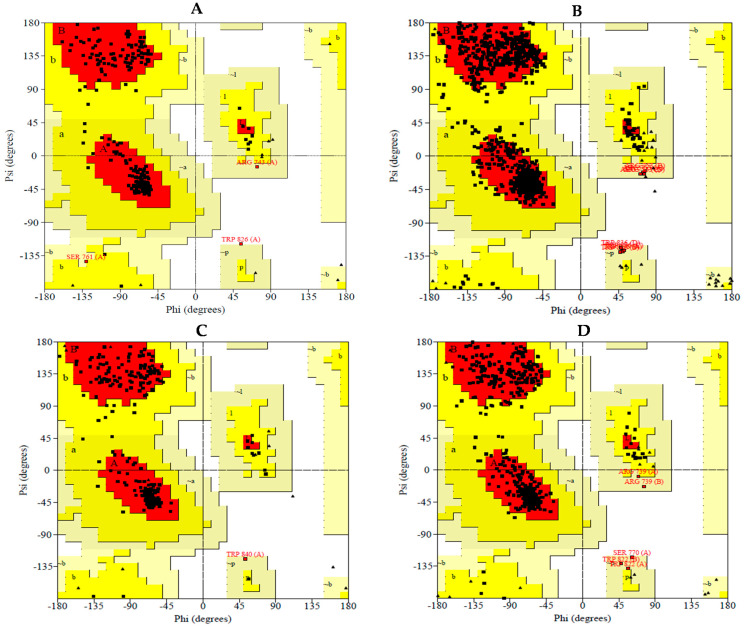
Ramachandran plots of EphB1 (**A**), EphB2 (**B**), EphB3 (**C**) and EphB4 (**D**); residues present in red-colored region signify the residues present in most favored region, additional allowed residues are found in the dark yellow-colored region, additional allowed residues are found in the light-yellow area and the disallowed residues are represented on white background color.

The crystal structures of the EphB isoforms have not been frequently utilized in previous studies; therefore, validation of their crystal structure was necessary. The PROCHECK server was utilized to produce Ramachandran plots for the four downloaded crystal structures, displaying allowed and disallowed regions (https://servicesn.mbi.ucla.edu/PROCHECK/, accessed on 21 May 2021) [30]. More than 90% residues should be in the most favored regions for the crystal structure to act as a standard model of good quality and reliability. The protein structures of EphB1, 2, 3 and 4 utilized in this study had 90.3%, 88.0%, 88.8%, and 89.0% residues, respectively, in the most favored region and 8.4%, 11.1%, 10.8%, and 9.8% residues, respectively, in the additional allowed regions (Figure 4). Hence, the structures selected for conducting the molecular docking study were predicted to be reliable, as more than 99% of the residues were found to be in the allowed regions.

#### 2.1.2. Generation of Virtual Combinatorial Library and Compounds Selection

To speed up the identification process and to decrease the cost, an in silico virtual combinatorial library was enumerated. This library was based on the hybridization-based strategy of incorporating the benzenesulfonamide moiety of compound **III** at position 4 of compound **II** to generate various derivatives. Additionally, to avoid any irreversible inhibition via the interaction with Cys717, a C-C linker was chosen to avoid any flexibility in the “tail” moiety which would prevent any possible interaction with Cys717. A virtual library was constructed using all aromatic and cyclic boronic acid derivatives available in our laboratory chemicals resulting in a virtual library of 34 hit compounds (Figure S1). The constructed library was subjected to a molecular docking simulation, using Maestro Schrodinger software, as a structure-based approach to facilitate and support the selection of compounds for synthesis. The virtual library was docked into the active site of the EphB3 crystal structure (PDB: 5L6P). GlideScore was used to rank the compounds based on their affinity to the target protein. The more negative the GlideScore the higher the affinity of the docked ligand to the target protein [31]. The top seven hits (1–7, Table 1) generated from docking of the virtual combinatorial library were chosen for further in-depth molecular docking study, MM-GBSA calculations, chemical synthesis, and biological evaluation. Each selected hit was given a synthetic code as follows: 1 (**4e**), 2 (**4c**), 3 (**4d**), 4 (**4a**), 5 (**4f**), 6 (**4b**), and 7 (**3a**).

### 2.2. Molecular Docking

Using the molecular docking to clarify the main protein-ligand interactions, the binding modes of the top seven generated hits from the combining virtual library was predicted in the EphB3 substrate-binding cavity. Docking of the active and selective co-crystallized inhibitor 6P8 (PDB: 5L6P [15]) demonstrated the ability of the modelling system to reproduce the reported crystal poses within 0.3 Å accuracy (data not shown). This, combined with the validation performed employing the PROCHECK server (Figure 4), offered assurance about the reliability of the chosen crystal structure in identifying potential EphB3 inhibitors.

Although each of the seven hits investigated by the molecular docking displayed a unique pattern of interaction with the active site of EphB3 active site, the sulfonamide and quinazoline moieties in all seven hit compounds formed an essential interaction, giving additional reassurance to the chosen design. Furthermore, all seven compounds interacted with Leu779, with the top two compounds (**4e** and **4c**) forming a hydrogen bond with Leu779 via the oxygen of the sulfonamide group (Table 1). Conversely, compounds **3a**, **4a**, **4b**, **4d** and **4f** formed π-Alkyl interactions with Leu779, implying that compounds **4e** and **4c** possess a higher affinity for EphB3. Compound **4e**, GlideScore of −7.30 kcal/mol, was predicted to interact with EphB3 through four typical hydrogen bonds, one carbon-hydrogen bond and three π-Alkyl interaction (Table 2). Although, the predicted binding mode of compound **4c** exhibited one less hydrogen bond when compared to compound **4e**, compound **4c** was able to interact with the active site residue through both; “head” and “tail” moieties indicating a better fit and predicting a higher biological activity. In summary, the predicted binding modes of compounds **3a** and **4a**–**4f** indicate their potential activity as EphB3 reversible inhibitors, marking them as good candidates for chemical synthesis and biological assay.

### 2.3. MM-GBSA Calculations

The free energy estimated using the MM-GBSA method is a useful tool to confirm the predicted potential affinity of a compound with its target. This is due to MM-GBSA calculations accounting for the effect of solvents on the complex’s stability. The lower the calculated binding free energy of a ligand protein complex, the more stable it is projected to be, as well as the higher the predicted activity and potency. As such, the MM-GBSA was calculated for compounds **3a** and **4a**–**f** as demonstrated in Table 3. Out of the top seven generated hits, compound **4c** displayed the highest free energy of −74.13 kcal/mol indicating its high stability when complexed with EphB3. Compound **4e** exhibited a slightly lowered free binding energy of −63.69 kcal/mol, while compounds **3a**, **4a**, **4b**, **4d** and **4f** displayed moderate to poor free binding energy of −53.14, −52.09, −35.79, −57.47 and −40.08 kcal/mol, respectively. Therefore, compounds **4e** and **4c** were predicted to possess the highest stability and predicted biological activity.

### 2.4. Chemical Synthesis

**Scheme 1:**
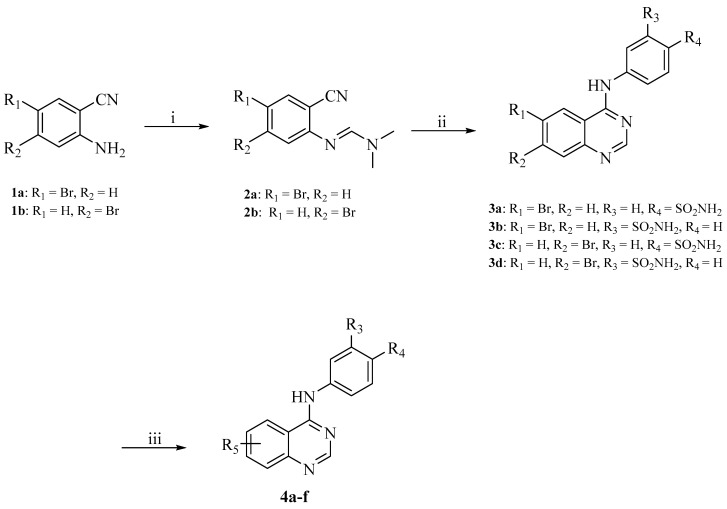
Reagents and conditions: (i) Dimethylformamide dimethyl acetal, 100 °C, 1.5 h; (ii) appropriately substituted aniline, glacial acetic acid, reflux, 2 h; (iii) appropriate boronic acid, Bis(di-tert-butyl(4-dimethylaminophenyl)phosphine)dichloropalladium(II), propylphosphonic anhydride, dioxane, 80 °C, 2 h.

The synthetic route used to obtain the desired target compounds **3a** and **4a**–**f** is outlined in Scheme 1. Refluxing of the commercially available 2-amino-5-bromobenzonitrile (**1a**) or 2-amino-4-bromobenzonitrile (**1b**) with dimethylformamide dimethyl acetal afforded compounds **2a**–**b**. Reacting compounds **2a**–**b** with the appropriate sulfonamide in glacial acetic acid under reflux resulted in the formation of compounds **3a**–**d**. The intermediates **3a**–**d** were then reacted with the appropriate boronic acid derivative via Suzuki cross-coupling using Bis(di-tert-butyl(4-dimethylaminophenyl)phosphine)dichloropalladium(II) as a coupling catalyst and the inorganic base, propylphosphonic anhydride, dissolved in dioxane and water at 80 °C under pressure in the presence of nitrogen to yield compounds **4a**–**f**. 

Different spectroscopic techniques including NMR, HRMS and HPLC were utilized to elucidate the chemical structures and the purity of the synthesized target compounds. The ^1^H NMR spectra of compounds **3a**–**d** are characterized by the appearance of two peaks at 7.20–7.50 ppm attributable to the sulfonamide NH_2_ and another peak at 10.10–10.30 ppm attributable to the NH of the amide group confirming the formation of compounds **3a**–**d** from their parent compounds **2a**–**b**. The success of the Suzuki cross-coupling reaction for compounds **4a**–**f** was confirmed by the appearance of characteristic peaks in the ^1^H NMR and ^13^C NMR spectra of the synthesized compounds. In particular, the appearance of a peak at 9.66 ppm in compound **4b** attributable to the carbonyl group or the appearance of a peak at 9.64 ppm in compound **4c** attributable to the hydroxy group confirmed the formation of these products. Similarly, compounds **4d** and **4e** displayed three characteristic peaks in the range of 2.30–2.40 and 2.40–3.50 ppm, respectively, validating the presence of the aliphatic hydrogens.

### 2.5. Biological Studies

#### 2.5.1. Assessment of the EphB3 Inhibitory Activity

**Figure 5:**
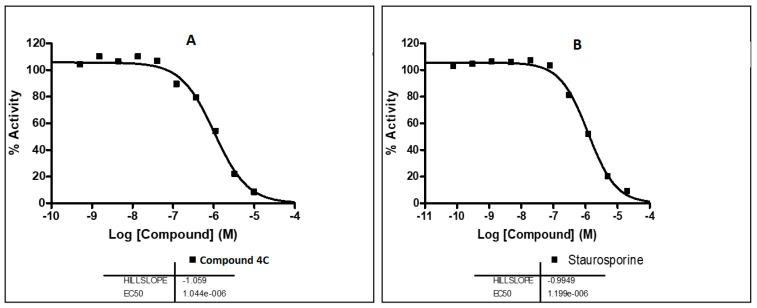
IC_50_ values of compound **4c** (**A**) and staurosporine (**B**) over EphB3 kinase.

The synthesized compounds were subjected to in vitro screening over EphB3 kinase. A single dose of 10 µM concentration of each target compound was used in EphB3 kinase inhibition assay, at 10 µM ATP, applying HotSpot^SM^ technology. Among the synthesized compounds, compound **4c** exhibited a potent EphB3 inhibitory activity of 93.27%. Consequently, it was selected for a further dose dependent assay to determine its IC_50_ value over EphB3 kinase. It exhibited a potent IC_50_ value of 1.04 µM (Figure 5). The results of the EphB3 inhibitory activity assay are demonstrated in Table 4.

#### 2.5.2. Assessment of the EGFR Inhibitory Activity

**Figure 6:**
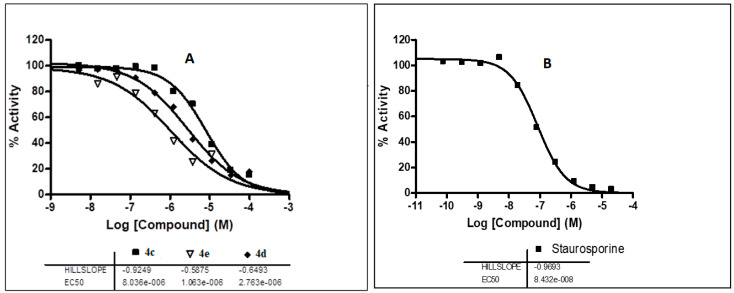
The IC_50_ values of compounds **4c**, **4e**, **4d** (**A**) and staurosporine over EGFR (**B**).

Many small-molecule inhibitors of EGFR kinase, epidermal growth factor receptor, are based on a quinazoline core, indicating that quinazolines have a high affinity for EGFR [32,33,34,35]. Accordingly, compounds possessing a quinazoline skeleton may have a potential to interact with the EGFR. Therefore, the synthesized compounds were tested over EGFR kinase at 10 µM concentrations in the presence of 10 M ATP (Table 5). Only compounds **4c**, **4d**, and **4e** displayed inhibition > 50%. Accordingly, the three compounds were selected for a further dose dependent assay to determine their IC_50_ values over EGFR. As shown in Figure 6, compounds **4c**, **4d**, and **4e** exhibited a modest EGFR inhibitory with IC_50_ values of 8.04, 2.76 and 1.06 µM, respectively, compared to the standard staurosporine (IC_50_ = 0.084 µM).

#### 2.5.3. Selectivity Assay of Compound **4c** over a Kinase Panel

To evaluate the selectivity and the kinase inhibition profile of the most potent EphB3 inhibitor of this new series (**4c**), an in vitro screening was carried out over a panel of cancer-related kinases and different isoforms of EphB kinase (Aurora A, Aurora B, CDK2/cyclin A, EphB1, EphB2, EphB4, ERBB2/HER2, and KDR/VEGFR2) in 10-dose IC_50_ mode with a 3-fold serial dilution starting at a concentration of 10 µM. The results of the selectivity study are demonstrated in Table 6. The results of the mini-kinase panel [36,37,38,39,40] indicate that among the four isoforms of EphB kinase, compound **4c** is selective toward EphB3 with more than 93% inhibition, while it exhibited a moderate activity against the other three isoforms EphB1, EphB2 and EphB4 with average inhibition of 58.89, 76.32, and 70.15%, respectively. Similarly, compound **4c** displayed poor to moderate inhibitory activity against the tested kinases with 67.86, 74.96, 67.75, 47.75, and 46.78% inhibition over Aurora A, CDK2, HER2, VEGFR2 and EGFR, respectively. 

A further dose-dependent assay was conducted to validate the selectivity of compound **4c** over the EphB isoforms. The assay assessed the IC_50_ values of **4c** over the EphB isoforms 1, 2 and 4 to be 5.86, 3.95 and 3.98 µM, respectively (Table 7). These values were found to be higher than the standard staurosporine which showed IC_50_ values of 30, 80 and 220 nM over EphB isoforms 1, 2 and 4, respectively. However, compound **4c** exhibited a 4-fold selectivity for EphB3 over EphB 2 and 4 isoforms and six-fold selectivity over the EphB1 isoform. Accordingly, it could be considered as a promising selective inhibitor for EphB3 isoform.

### 2.6. Molecular Docking Study 

#### 2.6.1. Molecular Docking Study for Compound **4c** over the EphB Isoforms

**Figure 7:**
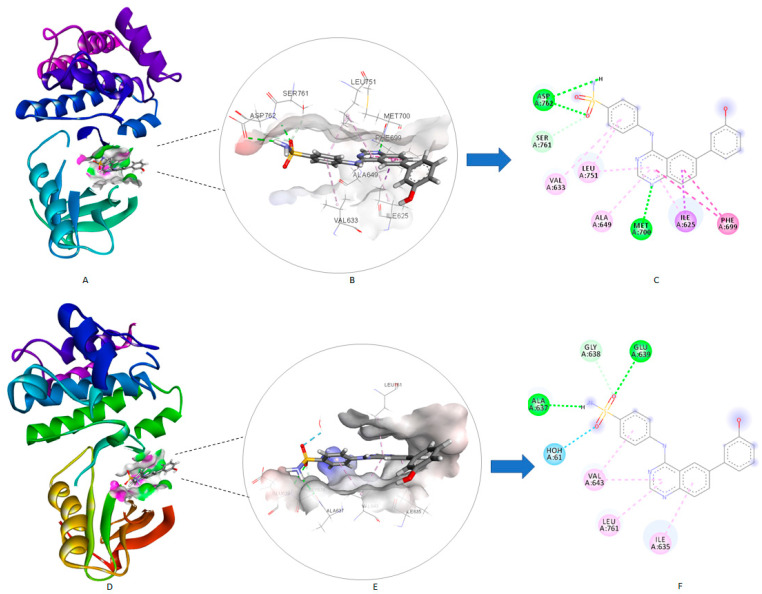
Docked complexes of compounds **4c** with EphB1 and EphB2 isoforms: (**A**) 3D model of the crystal structure of EphB1 with the compound **4c**; (**B**) 3D docking model of compound **4c** into EphB1 binding pocket; (**C**) 2D interaction diagram of compound **4c** with EphB1 active site; (**D**) 3D model of the crystal structure of EphB2 with compound **4c**; (**E**) 3D docking model of compound **4c** into EphB2 binding pocket; (**F**) 2D interaction diagram of compound **4c** with EphB2 active site.

**Figure 8:**
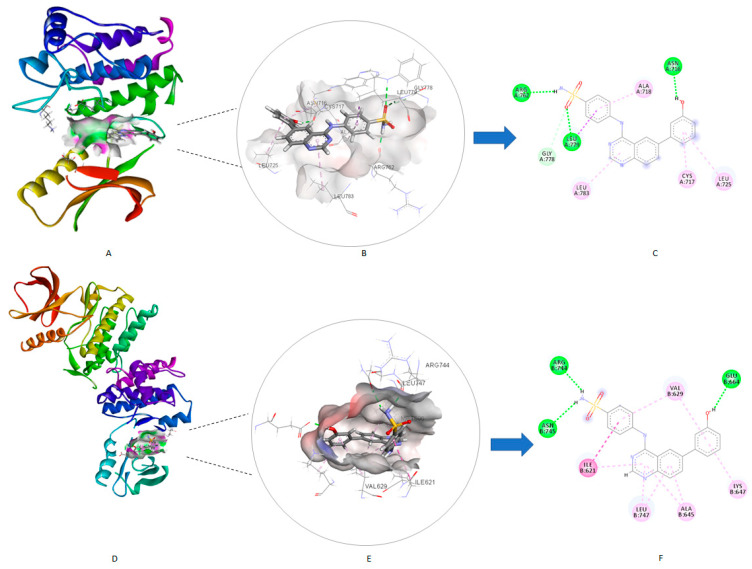
Docked complexes of compounds **4c** with EphB3 and EphB4 isoforms: (**A**) 3D model of the crystal structure of EphB3 with compound **4c**; (**B**) 3D docking model of compound **4c** into EphB3 binding pocket; (**C**) 2D interaction diagram of compound **4c** with EphB3 active site; (**D**) 3D model of the crystal structure of EphB4 with compound **4c**; (**E**) 3D docking model of compound **4c** into EphB4 binding pocket; (**F**) 2D interaction diagram of compound **4c** with EphB4 active site.

A molecular docking study was employed to elucidate the key protein-ligand interactions which may contribute to future structural modifications and SAR development. Docking of compound **4c** over EphB four isoforms revealed that the selectivity for EphB3 was due to two factors (the number of interactions formed with the active site and the conformation of **4c** inside the active site). The docking study demonstrated that compound **4c** was in a good conformation inside the active site of both EphB3 and 4 crystal structures, with both the “head” and “tail” regions of the compound participating in favorable interactions. Conversely, when compound **4c** was docked over EphB1 and 2 isoforms, only the sulfonamide of the “head” region was able to interact with the active site, leaving a large part of the compound outside the active site cavity, unable to form any favorable interactions (Figure 7). Compound **4c** formed four hydrogen bonds with the EphB1 isoform, involving Asp762, Ser761, and Met700 amino acids. However, the poor fit of the compound inside the active site may have contributed to its modest inhibition (58.89%). Compound **4c** was able to establish two hydrogen bonds with the EphB2 isoform. Conversely, compound **4c** established four hydrogen bonds with the amino acids Arg762, Gly778, Leu779, and Asn716 (Figure 8) when docked over the EphB3 isoform, which combined with a good conformation inside the active site explains its potent inhibition. In summary, out of the four isoforms of EphB, compound **4c** showed both; a good fit with the active site and several favorable interactions over the EphB3 isoform, which help to explain its potent IC_50_ value of 1.04 µM. On the contrary, the lack of a good fit or the presence of favorable interactions led to weaker binding profile over the EphB1, 2, and 4 isoforms.

#### 2.6.2. Molecular Docking Study for Compound **4c** over the Tested Kinase Panel

**Figure 9:**
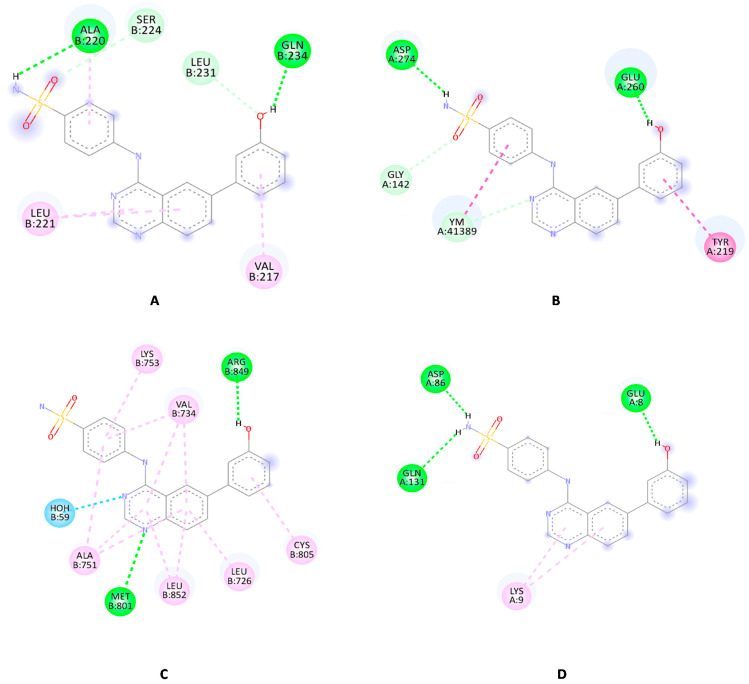
The 2D interaction pattern of compound **4c** in the binding sites of Aurora A (**A**), VEGFR2 (**B**), HER2 (**C**) and CDK2 (**D**). Favorable interactions are color coded as follows: green, hydrogen bonds; dark pink, π–π stacking interactions; purple, π–sigma interactions; light green, unconventional carbon–hydrogen bonds.

A molecular docking study was carried out for compound **4c** over the tested kinases to clarify the key protein-ligand interactions and get molecular insights for its modest biological activity over these kinases. Compound **4c** established two hydrogen bonds with ALA220 and GLN 234 amino acid residues of the Aurora A binding site (Figure 9A). It was able to form three further weak interactions with the active site of Aurora A which explains its moderate inhibitory activity (67.86%). Similarly, it formed two hydrogen bonds with VEGFR2 and HER2 (Figure 9B and Figure 9C, respectively) with inhibitory activity of 47.94% and 67.75%, respectively. Conversely, it established three hydrogen bonds with CDK2 (Figure 9D), which may clarify the increase in its inhibitory activity against CDK2 (74.96%). 

## 3. Materials and Methods

### 3.1. Protein Preparation

The crystal structures of EphB1 (PDB code: 6UMW, Resolution: 1.98 Å) [41], EphB2 (PDB code: 2HEN, Resolution: 2.60 Å) [42], EphB3 (PDB ID: 5L6P, Resolution: 2.26 Å) [15], EphB4 (PDB ID: 3ZEW, Resolution: 2.50 Å) [43], VEGFR2 (PDB code: 3VHE, Resolution: 1.55 Å) [44], Aurora A (PDB code: 2X6E, Resolution: 3.35 Å) [45], HER2 (PDB code: 3PP0, Resolution: 2.25 Å) [46] and CDK2 (PDB code3PY0, Resolution: 1.75 Å) [47] were downloaded from protein data bank (www.pdb.org, accessed on 18 April 2021). To ensure the validity of the downloaded crystal structures, PROCHECK server was employed to generate a Ramachandran plot for all four downloaded crystal structures to ensure their validity [30]. The preparation wizard of Schrodinger 2021 suite package was used to prepare the downloaded Protein structures at the default setting with pH = 7.4. The ligands were sketched employing ChemDraw Professional 17.0 (PerkinElmer, Inc., Waltham, MA, USA) and saved in the structure data file format which was then uploaded to the Ligprep module. Subsequently, the Ligprep module of Schrodinger was applied in the ligand preparation step to further optimizing the geometry. The generated docking models were validated by re-docking of the X-ray ligand, thus confirming the reproducibility of the results (data not shown). The minimized ligands were docked into the appropriate crystal structure binding site using Glide’s standard precision module to generate ten poses for the docked ligand. Discovery Studio visualizer Client 2021 package (Dassault Systèmes, Vélizy-Villacoublay, France) was used for the visualization and illustration of the generated ligand-protein complexes.

### 3.2. Molecular Mechanics-Generalized Born Surface Area (MM-GBSA) Calculations 

The binding free energy of the examined protein-ligand complexes was calculated using the MM-GBSA approach, which combined molecular mechanics (MM) force fields with a generalized Born and surface area continuum (implicit) solvation solvent model. OPLS-2005 force field, VSGB solvent model, and rotamer search algorithms were applied in the MM-GBSA computation [48]. The MM-GBSA was calculated using the prime module of the Schrodinger suite (Prime, Schrödinger, LLC, New York, NY, USA) according to the equation below:

ΔG = E_complex (minimized) − [E_ligand (minimized) + E_receptor (minimized)]

The MM-GBSA was calculated using the default configuration, which rendered all protein atoms rigid while allowing ligand atoms to be in a relaxed state.

### 3.3. Chemistry

The purchased solvents and reagents were used without further purification in the conducted experiments. Varian 400 MHz spectrometer (Varian Medical Systems, Inc., Palo Alto, CA, USA) was used to record the ^1^H NMR spectra with chemical shifts being recorded in parts per million (ppm) and coupling constants in Hz, as reported previously [49]. The high-resolution electrospray ionization mass spectrometry (HR-ESIMS) data were recorded on a JMS-700 mass spectrometer (Jeol, Japan) or by HR-ESIMS data recorded on a G2 QTOF mass spectrometer (Thermo Scientific™, Waltham, MA, USA). The reactions were monitored using TLC on 0.25 mm silica plates (E. Merck, Darmstadt, Germany; silica gel 60 F_254_). The purity of the products was determined by reversed-phase high performance liquid chromatography (RP-HPLC) utilizing Waters Corp. HPLC system equipped with a UV detector set at 254 nm (Milford, MA, USA). The mobile phases used were: (A) H_2_O containing 0.05% TFA and (B) CH_3_CN. HPLC employed a YMC Hydrosphere C18 (HS-302) column (5 μM particle size, 12 nm pore size) that was 4.6 mm in diameter × 150 mm in size, with a flow rate of 1.0 mL/min. The final compounds purity was assessed using either a gradient of 75% B or 100% B in 30 min. A Fisherbrand digital melting point apparatus was used to measure the melting points.

#### 3.3.1. General Procedure for Synthesis of Compounds **2a**–**b**

A mixture containing 1 mmol of 2-amino-5-bromobenzonitrile (1a) or 2-amino-4-bromobenzonitrile (1b) and dimethylformamide dimethyl acetal (10 mL) was stirred at reflux for 1.5 h. The mixture was cooled to room temperature and refrigerated. The solid was filtered, washed with several portions of ether, and dried to yield the desired compound which was used in the next step without further purification [50]. 

#### 3.3.2. General Procedure for Synthesis of Compounds **3a**–**d**

A mixture of (E)-N′-(5-bromo-2-cyanophenyl)-N,N-dimethylformimidamide (**2a**) or (E)-N′-(4-bromo-2-cyanophenyl)-N,N-dimethylformimidamide (**2b**) and the appropriate aniline derivative (1 eq.) in glacial acetic acid (15.0 mL) was refluxed for 2 h. The acetic acid was evaporated and the solid was washed with water and diethyl ether and dried to afford the titled compounds which were used in the next step without further purification [50].

##### 4-((6-Bromoquinazolin-4-yl)amino)benzenesulfonamide (**3a**) 

Off-white solid, yield: 76%, mp: 322.2–324.5 °C, HPLC purity: 15 min, 99.45%, ^1^H NMR (400 MHz, DMSO-d6) δ 10.09 (s, 1H), 8.88 (s, 1H), 8.69 (s, 1H), 8.09–8.03 (d, J = 8.4 Hz, 2H), 8.03–7.98 (d, J = 8.8 Hz, 1H), 7.85–7.79 (d, J = 8.4 Hz, 2H), 7.78–7.74 (d, J = 8.8 Hz, 1H), 7.27 (s, 2H). ^13^C NMR (100 MHz, DMSO-d_6_) δ 157.10, 155.03, 149.04, 142.44, 139.05, 136.75, 130.57, 126.77, 125.91, 121.93, 119.58, 116.93. HRMS (ESI) *m*/*z* calculated for C_14_H_11_BrN_4_O_2_S: [M + H]^+^: 378.9859. Found: 378.9856.

##### 3-((6-Bromoquinazolin-4-yl)amino)benzenesulfonamide (**3b**) 

Off-white solid, yield: 90%, mp: 325.0–326.4 °C, HPLC purity: 12 min, 95.11%, ^1^H NMR (400 MHz, DMSO-d_6_) δ 10.12 (s, 1H), 8.92 (d, J = 2.0 Hz, 1H), 8.68 (s, 1H), 8.38 (s, 1H), 8.20 (m, 1H), 8.02 (dd, J = 8.0, 2.0 Hz, 1H), 7.77 (d, J = 8.0 Hz, 1H), 7.63–7.58 (m, 2H), 7.42 (s, 2H). HRMS (ESI) *m*/*z* calculated for C_14_H_11_BrN_4_O_2_S: [M + H]^+^: 378.9859. Found: 378.9850.

##### 4-((7-Bromoquinazolin-4-yl)amino)benzenesulfonamide (**3c**)

Off white solid, yield: 18%, mp: 320.3–322.1 °C, ^1^H NMR (400 MHz, DMSO-d_6_) δ 10.16 (s, 1H), 8.68 (s, 1H), 8.56 (d, J = 9.2 Hz, 1H), 8.11–8.02 (m, 3H), 7.85 (m 3H), 7.29 (s, 2H). ^13^C NMR (100 MHz, DMSO-d_6_) δ 158.15, 155.58, 150.68, 142.39, 139.29, 130.13, 129.90, 127.51, 126.76, 125.84, 122.21.

##### 3-((7-Bromoquinazolin-4-yl)amino)benzenesulfonamide (**3d**)

White solid, yield: 97%, mp: 318.6–320.5 °C, ^1^H NMR (400 MHz, DMSO-d_6_) δ 10.18 (s, 1H), 8.66 (s, 1H), 8.56 (d, J = 8.8 Hz, 1H), 8.37 (s, 1H), 8.20–8.13 (m, 1H), 8.03 (d, J = 2.0 Hz, 1H), 7.86 (dd, J = 8.8, 2.0 Hz, 1H), 7.61 (t, J = 6.6 Hz, 2H), 7.42 (s, 2H). ^13^C NMR (100 MHz, DMSO-d_6_) 158.15, 155.82, 151.27, 144.89, 139.85, 130.07, 127.34, 125.74, 119.53, 114.51.

#### 3.3.3. General Procedure for Synthesis of Compounds **4a**–**f**

Compounds **3a**–**d** were allowed to react with the appropriate boronic acid derivative via the Suzuki cross-coupling reaction in the presence of the coupling catalyst bis(di-tert-butyl(4-dimethylaminophenyl)phosphine)dichloropalladium(II) and an inorganic base propylphosphonic anhydride. The reaction mixture was dissolved in dioxane and water and then heated to 80 °C for 2 h under pressure in the presence of nitrogen to produce compounds **4a**–**f** [51].

##### 4-((6-(Pyridin-3-yl)quinazolin-4-yl)amino)benzenesulfonamide (**4a**)

Off-white solid, yield: 22%, mp: 315.1–316.2 °C, HPLC purity: 8 min, 97.84%, ^1^H NMR (400 MHz, DMSO-d_6_) δ 10.13 (s, 1H), 9.13 (s, 1H), 8.93 (s, 1H), 8.71–8.59 (m, 2H), 8.27 (d, J = 8.0 Hz, 2H), 8.07 (d, J = 8.0 Hz, 2H), 7.92 (d, J = 8.0 Hz, 1H), 7.84 (d, J = 8.0 Hz, 2H), 7.61–7.53 (m, 1H), 7.29 (s, 2H). ^13^C NMR (100 MHz, DMSO-d_6_) δ 158.10, 154.95, 149.93, 149.48, 149.38, 148.71, 148.49, 142.66, 139.09, 135.66, 134.97, 132.37, 129.33, 126.90, 126.68, 124.54, 122.17, 121.55, 115.89. HRMS (ESI) *m*/*z* calculated for C_19_H_15_N_5_O_2_S: [M + H]^+^: 378.1019. Found: 378.1012.

##### 4-((6-(5-Formylfuran-2-yl)quinazolin-4-yl)amino)benzenesulfonamide (**4b**)

Off-white solid, yield: 22%, mp: 263.4–236.9 °C, HPLC purity: 13 min, 95.58%, ^1^H (400 MHz, DMSO-d_6_) δ10.36 (s, 1H), 9.66 (s, 1H), 9.03 (s, 1H), 8.67 (s, 1H), 8.33 (d, J = 8.0 Hz, 1H), 8.05 (d, J = 8.0 Hz, 2H), 7.90 (d, J = 8.0 Hz, 1H), 7.84 (d, J = 8.0 Hz, 2H), 7.75 (d, J = 4.0 Hz, 1H), 7.44 (d, J = 4.0 Hz, 1H), 7.30 (s, 2H). ^13^C NMR (100 MHz, DMSO-d_6_) δ 178.41, 158.26, 157.97, 155.49, 152.60, 150.76, 139.23, 130.37, 129.39, 127.06, 126.73, 122.45, 120.01, 115.97, 110.50, 40.36, 40.09, 39.95, 39.67. HRMS (ESI) *m*/*z* calculated for C_19_H_14_N_4_O_4_S: [M + H]^+^: 395.0809. Found: 395.0805.

##### 4-((6-(3-Hydroxyphenyl)quinazolin-4-yl)amino)benzenesulfonamide (**4c**) 

Off-white solid, yield: 45%, mp: 313.2–315.1 °C, HPLC purity: 13 min, 95.10%, ^1^H NMR (400 MHz, DMSO-d_6_) δ 10.16 (s, 1H), 9.64 (s, 1H), 8.80 (s, 1H), 8.65 (s, 1H), 8.13 (dd, J = 8.0, 1.6 Hz, 1H), 8.07 (d, J = 8.0 Hz, 2H), 7.85 (m, 3H), 7.29 (m, 5H), 6.84 (dd, J = 8.0, 1.6 Hz, 1H). ^13^C NMR (100 MHz, DMSO-d_6_) δ 158.37, 158.10, 154.58, 149.62, 142.75, 140.99, 139.01, 138.94, 132.49, 130.49, 128.89, 126.72, 122.07, 120.81, 118.46, 115.84, 115.37, 114.53. HRMS (ESI) *m*/*z* calculated for C_20_H_16_N_4_O_3_S: [M + H]^+^: 393.1016. Found: 393.1007.

##### 4-((7-(4-(Morpholinomethyl)phenyl)quinazolin-4-yl)amino)benzenesulfonamide (**4d**)

Off-white crystals, yield: 24%, mp: 228.1–229.8 °C, HPLC purity: 10 min, 96.63%, ^1^H NMR (400 MHz, DMSO-d_6_) δ 10.10 (s, 1H), 8.72–8.66 (m, 2H), 8.13 (d, J = 8.8 Hz, 2H), 8.08 (s, 1H), 8.04 (d, J = 8 Hz, 1H), 7.86 (t, J = 8 Hz, 4H), 7.49 (d, J = 8 Hz, 2H), 7.29 (s, 2H) 3.61–3.56 (m, 4H), 3.58 (s, 1H), 3.55 (s, 1H), 2.38 (s, 4H). ^13^C NMR (100 MHz, DMSO-d_6_) δ 157.87, 155.21, 155.18, 150.79, 144.89, 142.85, 138.96, 138.80, 137.68, 130.22, 130.03, 127.64, 127.49, 126.83, 125.87, 125.22, 125.08, 124.33, 121.83, 114.65, 66.67, 62.46, 53.66. HRMS (ESI) *m*/*z* calculated for C_25_H_25_N_5_O_3_S: [M + H]^+^: 476.1751. Found: 476.1745.

##### 3-((7-(3-(Morpholinomethyl)phenyl)quinazolin-4-yl)amino)benzenesulfonamide (**4e**)

Off-white solid, yield: 32%, mp: 267.5–269.0 °C, HPLC purity: 10 min, 99.15%, ^1^H NMR (400 MHz, DMSO-d_6_) δ 10.11 (s, 1H), 8.72–8.64 (m, 2H), 8.43 (s, 1H), 8.22 (m, 1H), 8.08–8.01 (m, 2H), 7.87 (d, J = 8.0 Hz, 2H), 7.60 (m, 2H), 7.48 (d, J = 8.2 Hz, 2H), 7.41 (s, 2H), 3.62–3.58 (m, 4H), 3.55 (s, 2H), 2.40 (s, 4H). ^13^C NMR (100 MHz, DMSO-d_6_) δ 157.89, 155.27, 150.79, 144.90, 142.85, 138.83, 137.69, 130.30, 127.69, 127.42, 126.90, 126.61, 125.70, 125.25, 125.05, 124.38, 124.13, 121.88, 114.66, 66.68, 62.46, 53.66, 49.06. HRMS (ESI) *m*/*z* calculated for C_25_H_25_N_5_O_3_S: [M + H]^+^: 476.1751. Found: 476.1744.

##### 3-((6-(Pyridin-3-yl)quinazolin-4-yl)amino)benzenesulfonamide (**4f**)

Off-white solid, yield: 26%, mp: 320.0–321.5 °C, HPLC purity: 8 min, 98.93%, ^1^H NMR (400 MHz, DMSO-d_6_) δ 10.16 (s, 1H), 9.15 (s, 1H), 8.97 (s, 1H), 8.70–8.64 (m, 2H), 8.37 (s, 1H), 8.33–8.26 (m, 2H), 8.24–8.16 (m, 1H), 7.94 (d, J = 8.2 Hz, 1H), 7.65–7.57 (m, 3H), 7.42 (s, 2H). ^13^C NMR (100 MHz, DMSO-d_6_) δ 158.18, 155.06, 149.87, 149.40, 148.55, 144.91, 139.94, 135.59, 134.90, 132.32, 129.62, 129.19, 125.68, 124.39, 121.44, 121.19, 119.48, 115.79. HRMS (ESI) *m*/*z* calculated for C_19_H_15_N_5_O_2_S: [M + H]^+^: 379.1019. Found: 378.1010.

### 3.4. In Vitro Kinase Assay

The screening of the tested compounds was performed using the Kinase HotSpot^SM^ service from Reaction Biology Co. (http://www.reactionbiology.com, accessed on 25 September 2021). The HotSpot assay platform contains specific kinase/substrate pairs along with required cofactors. Base reaction buffer: 20 mM Hepes (pH 7.5), 10 mM MgCl_2_, 1 mM EGTA, 0.02% Brij35, 0.02 mg/mL BSA, 0.1 mM Na_3_VO_4_, 2 mM DTT, 1% DMSO. The tested compounds were dissolved in 100% DMSO to specific concentration. The serial dilution was conducted by Integra Viaflo Assist in DMSO (Sigma-Aldrich, Burlington, MA, USA). The reaction mixture containing the examined compound and 33P-ATP was incubated at room temperature for 2 h, and radioactivity was detected by filter-binding method. Kinase activity data were expressed as the percent remaining kinase activity in test samples compared to vehicle (dimethyl sulfoxide) reactions. IC_50_ values and curve fits were obtained using Prism (GraphPad Software, San Diego, CA, USA). 

## 4. Conclusions

As an efficient approach to speed up the identification process of new EphB3 inhibitors, a virtual library was constructed based on a structure-based design and was subjected to an in-depth molecular docking study. The docking study was used to select the most promising compounds for MM-GBSA calculations, chemical synthesis, and biological evaluation. The MM-GBSA calculations predicted compound **4c** to possess the highest stability with a binding free energy (−74.13 Kcal/mol). The kinase assays confirmed the potency of compound **4c** as a potent EphB3 inhibitor (IC_50_ = 1.04 µM). Furthermore, a kinase panel assay composed of EGFR, Aurora A, Aurora B, CDK2/cyclin A, EphB1, EphB2, EphB4, ERBB2/HER2, and KDR/VEGFR2 revealed that compound **4c** has a promising selectivity profile, with a four-fold selectivity for EphB3 over EphB2 and four isoforms and six-fold selectivity over the EphB1 isoform. These results indicate that compound **4c** is a promising selective EphB3 inhibitor. Accordingly, it may be a useful tool to enhance the understanding of EphB3 biological role and pave the way toward identification of more potent and selective inhibitors. Additionally, the results show the validity of the constructed experimental model to predict novel EphB3 inhibitors. 

## Figures and Tables

**Table 1:** The hits generated from the virtual combinatorial library and their respective GlideScores.

Hit Cpd	GlideScore (kcal/mol)	Hit Cpd	GlideScore (kcal/mol)
1	−7.30	18	−5.78
2	−7.16	19	−5.63
3	−6.65	20	−5.50
4	−6.47	21	−5.49
5	−6.23	22	−5.47
6	−6.21	23	−5.46
7	−6.20	24	−5.35
8	−6.18	25	−5.35
9	−6.13	26	−5.32
10	−6.12	27	−5.31
11	−6.11	28	−5.16
12	−6.10	29	−5.09
13	−6.07	30	−4.80
14	−6.06	31	−4.59
15	−5.90	32	−4.49
16	−5.84	33	−4.24
17	−5.84	34	−4.22

**Table 2:** Molecular docking study results of the top seven hits generated from the virtual combinatorial library.

Cpd	GlideScore (kcal/mol)	2D Diagram	Amino Acids	Binding Group	Molecular Interactions
**3a**	−6.20	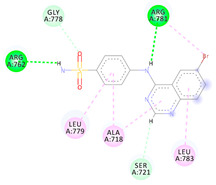	Arg762 Arg781 Ser721 Leu779 Ala718 Leu783	NH (SO_2_NH_2_) NH (amide) Benzene Benzene Benzene Benzene	H-bond H-bond π-donor H-bond π-Alkyl π-Alkyl π-Alkyl
**4a**	−6.47	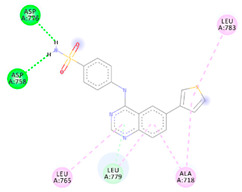	Asp776 Asp758 Leu779 Leu765 Ala718 Leu783	NH (SO_2_NH_2_) NH (SO_2_NH_2_) Benzene Benzene Benzene Benzene	H-bond H-bond C-H-bond π-Alkyl π-Alkyl π-Alkyl
**4b**	−6.21	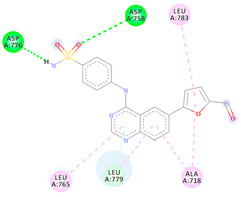	Asp776 Asp758 Leu779 Leu765 Ala718 Leu783	NH (SO_2_NH_2_) O (SO_2_NH_2_) Benzene Benzene Benzene Benzene	H-bond H-bond π-donor H-bond π-Alkyl π-Alkyl π-Alkyl
**4c**	−7.16	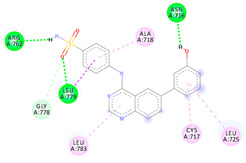	Arg762 Asn716 Leu779 Gly778 Ala718 Leu783 Cys717 Leu725	NH (SO_2_NH_2_) OH O (SO_2_NH_2_) O (SO_2_NH_2_) Benzene Benzene Benzene Benzene	H-bond H-bond H-bond C-H-bond π-Alkyl π-Alkyl π-Alkyl π-Alkyl
**4d**	−6.65	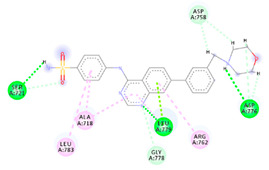	Ser721 Leu779 Asp776 Gly778 Asp758 Leu783 Arg762	NH (SO_2_NH_2_) H (morpholine) H (morpholine) N (quinazoline) H (morpholine) Benzene Benzene	H-bond H-bond H-bond C-H-bond C-H-bond π-Alkyl π-Alkyl
**4e**	−7.30	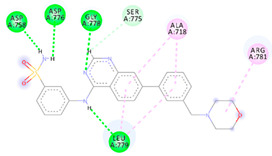	Asp758 Asp776 Gly778 Leu779 Ser775 Ala718 Arg781	NH (SO_2_NH_2_) NH (SO_2_NH_2_) N (quinazoline) NH (amide) H (quinazoline) Benzene Benzene	H-bond H-bond H-bond H-bond C-H-bond π-Alkyl π-Alkyl
**4f**	−6.23	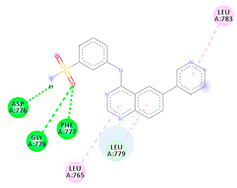	Asp776 Gly778 Phe777 Leu779 Leu765 Leu783	NH (SO_2_NH_2_) O (SO_2_NH_2_) O (SO_2_NH_2_) Benzene Benzene Benzene	H-bond H-bond H-bond π-donor H-bond π-Alkyl π-Alkyl

**Table 3:** MM-GBSA calculations for compounds **3a** and **4a**–**f**.

Cpd	MM-GBSA (kcal/mol)
**3a**	−53.14
**4a**	−52.09
**4b**	−35.79
**4c**	−74.13
**4d**	−57.47
**4e**	−63.69
**4f**	−40.08

**Table 4:** % Inhibition values of the synthesized compounds **3a** and **4a**–**f** over EphB3 kinase at 10 µM concentration.

Cpd	Structure	Average % Enzyme Inhibition at 10 µM (Relative to DMSO Control)
**3a**	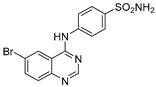	26.97 ± 0.24
**4a**	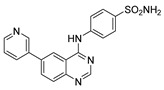	25.81 ± 0.24
**4b**	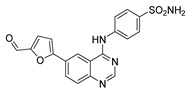	26.71 ± 0.45
**4c**	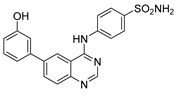	93.27 ± 0.06
**4d**	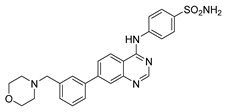	18.86 ± 1.09
**4e**	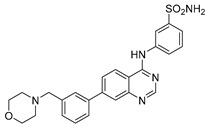	−21.35 ± 0.09
**4f**	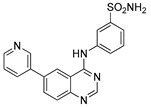	23.48 ± 5.36

**Table 5:** % Inhibition values of the synthesized compounds **3a** and **4a**–**f** over EGFR kinase at a single dose concentration of 10 µM.

Cpd	Average % Enzyme Inhibition (Relative to DMSO Controls) at 10 µM
**3a**	−13.16 ± 4.31
**4a**	0.56 ± 4.32
**4b**	25.25 ± 1.25
**4c**	46.78 ± 0.54
**4d**	65.76 ± 0.98
**4e**	63.28 ± 0.21
**4f**	4.57 ± 2.16
Staurosporine	-

**Table 6:** % Inhibition values of compound **4c** over a panel of different kinases.

Kinase	Average % Enzyme Inhibition (Relative to DMSO Controls) at 10 µM
Aurora A	67.86 ± 4.93
CDK2/cyclin A	74.96 ± 2.07
EphB1	58.89 ± 1.98
EphB2	76.32 ± 0.61
EphB3	93.27 ± 0.06
EphB4	70.15 ± 3.64
ERBB2/HER2	67.75 ± 0.99
KDR/VEGFR2	±0.66

**Table 7:** IC_50_ values of compound **4c** over the EphB isoforms.

Kinase	4c IC_50_ (µM)	Staurosporine IC_50_ (µM)
**EphB1**	5.86	0.03
**EphB2**	3.95	0.08
**EphB3**	1.04	1.20
**EphB4**	3.98	0.22

## Data Availability

Data are contained within the article.

## Supplementary Materials

The following are available online at https://www.mdpi.com/article/10.3390/ph14121247/s1, Figure S1: The chemical structures of the generated virtual library, ^1^H NMR, ^13^C NMR, HPLC, and HRMS data of compounds **3a**–**d** and **4a**–**f**.
