# Supplementary material for: Hit Identification of a Novel Quinazoline Sulfonamide as a Promising EphB3 Inhibitor: Design, Virtual Combinatorial Library, Synthesis, Biological Evaluation, and Docking Simulation Studies
Source: Pharmaceuticals (Basel). 2021 Nov 30;14(12):1247. doi: 10.3390/ph14121247 (PMC8708751; doi:10.3390/ph14121247)
Supplement: Supplementary file 1 [file pharmaceuticals-14-01247-s001.zip › pharmaceuticals-1478553-supplementary.pdf]

Supplementary material

Hit Identification of a Novel Quinazoline Sulfonamide as a promising EphB3 inhibitor: Design, Virtual Combinatorial Library, Synthesis, Biological Evaluation, and Docking Simulation Studies

Contents

1. Figure S1. The chemical structures of the generated virtual library
2. ^{1}HNMR and ^{13}CNMR data of compounds **3a–d** and **4a–f**
3. Representative HPLC purity data of the compounds **3a–d** and **4a–f**
4. Representative HRMS data of the compounds **3a–d** and **4a–f**

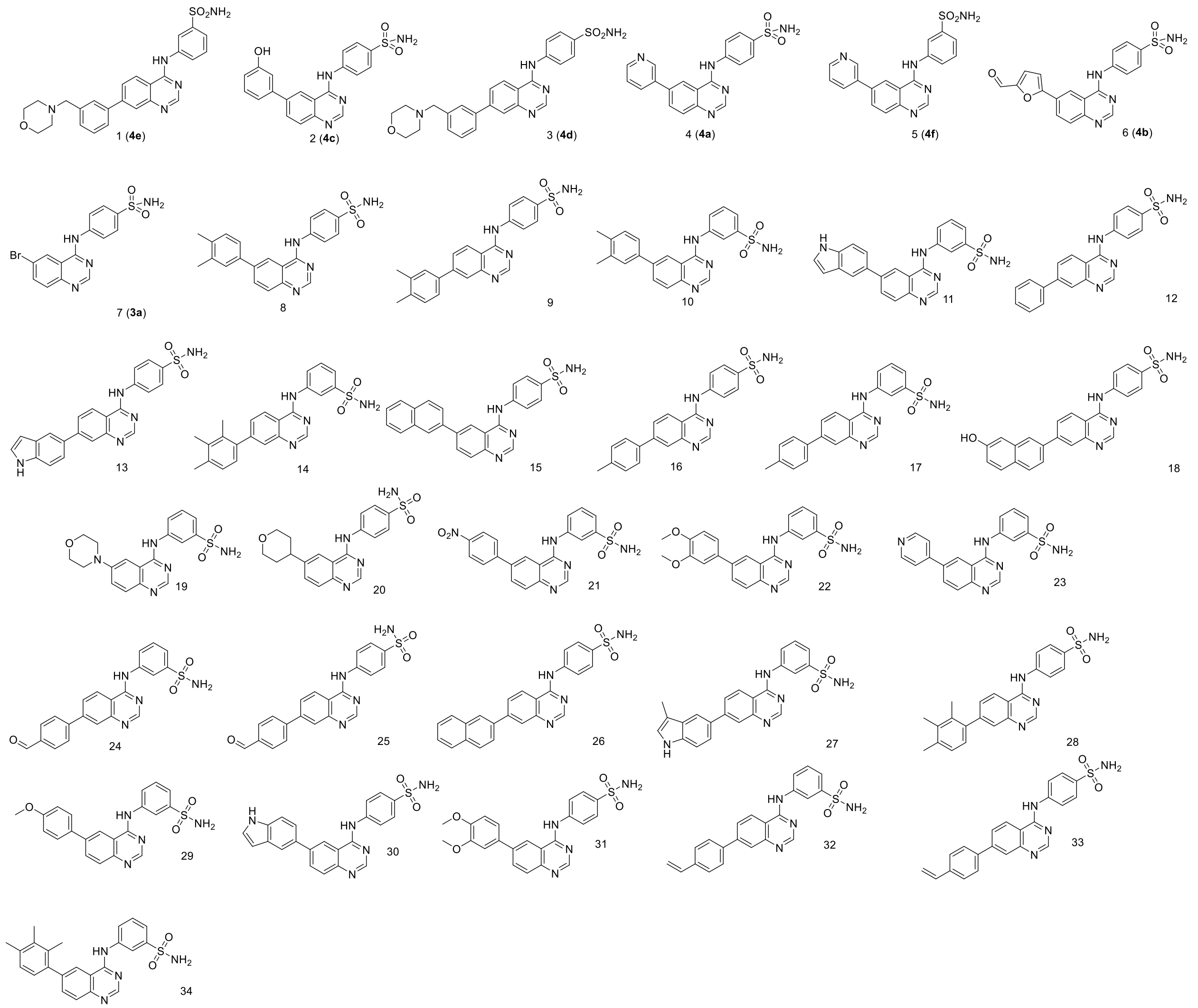

1. Figure S1. The chemical structures of the generated virtual library compounds

2. ^{1}HNMR and ^{13}CNMR data of compounds 3a–d and 4a–f

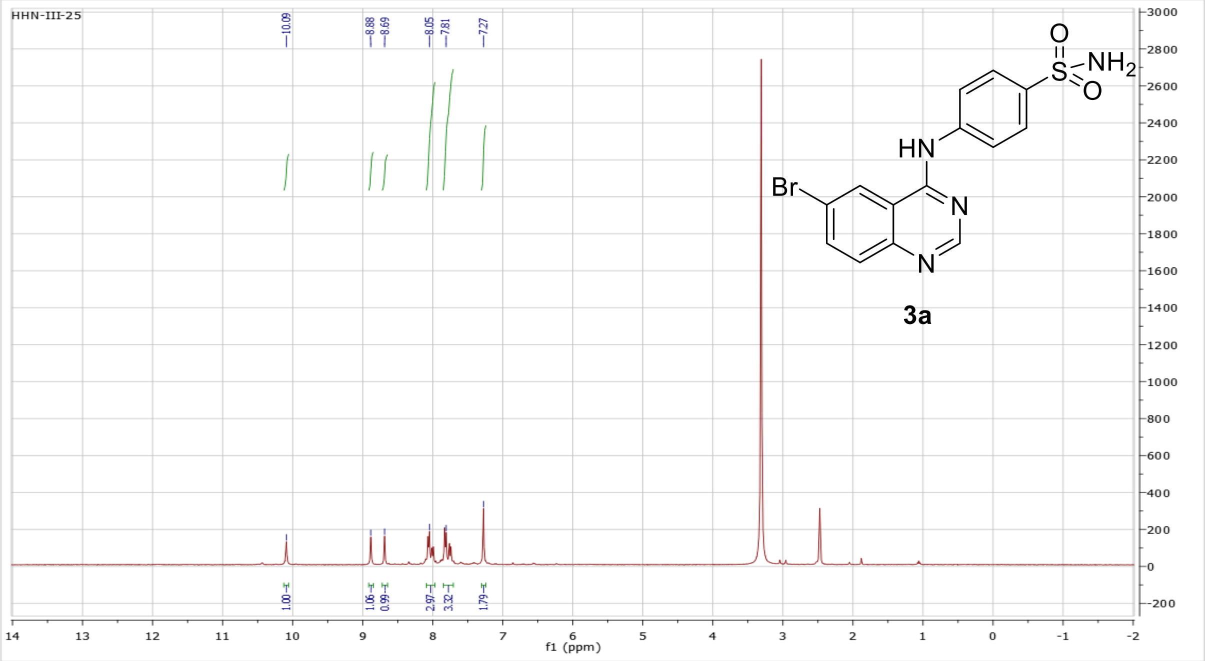

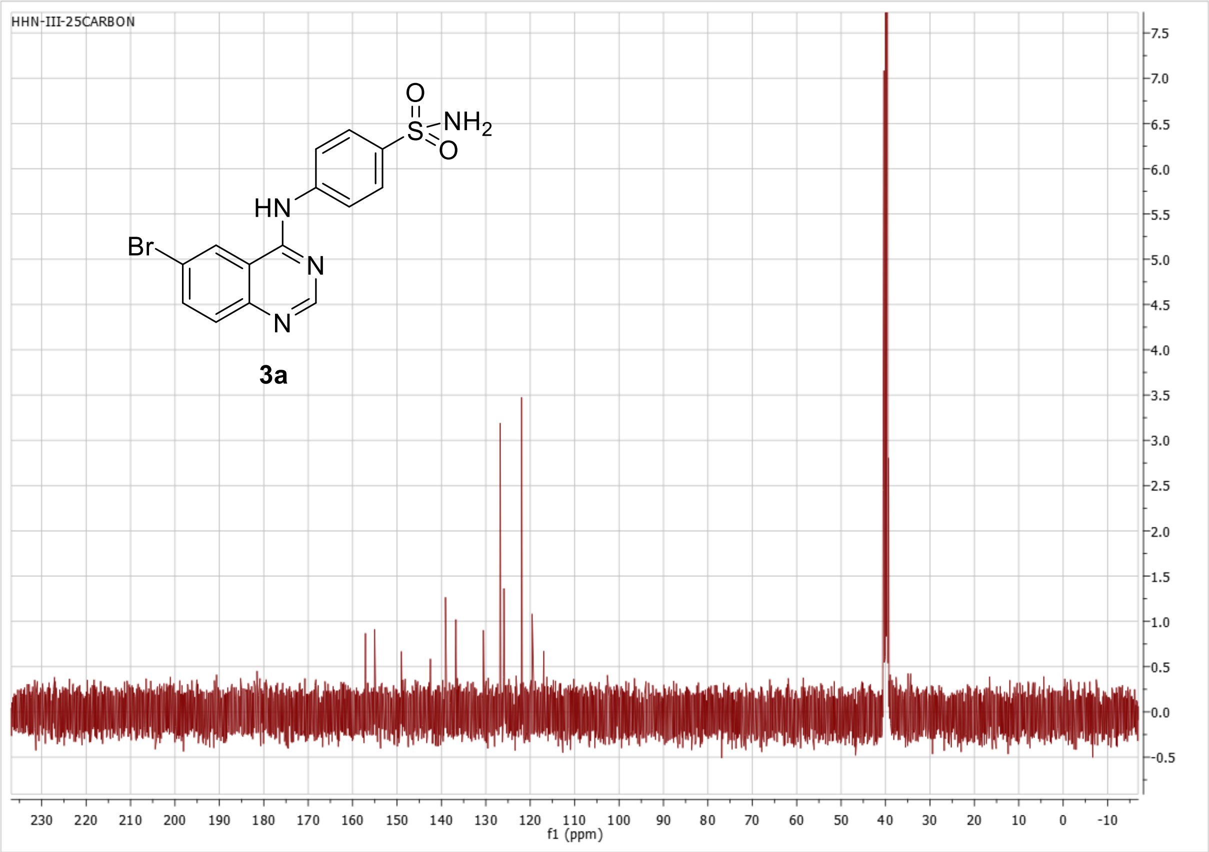

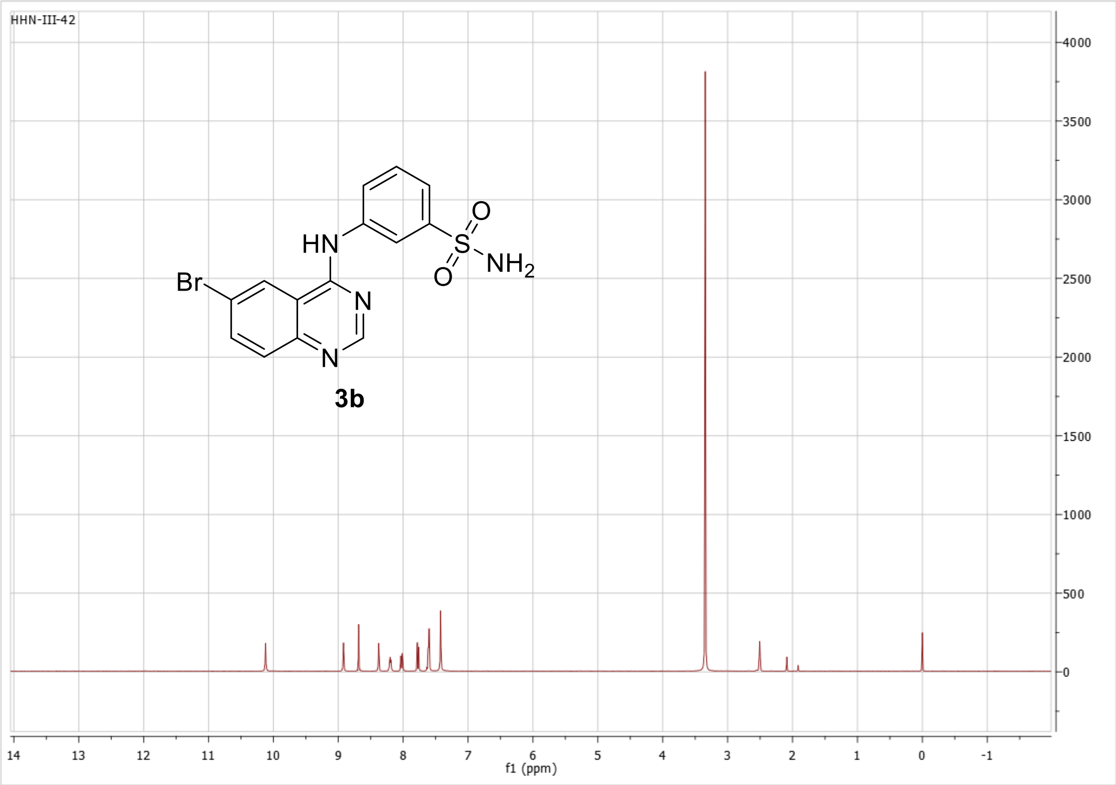
HHN-III-42
3b
f1 (ppm)

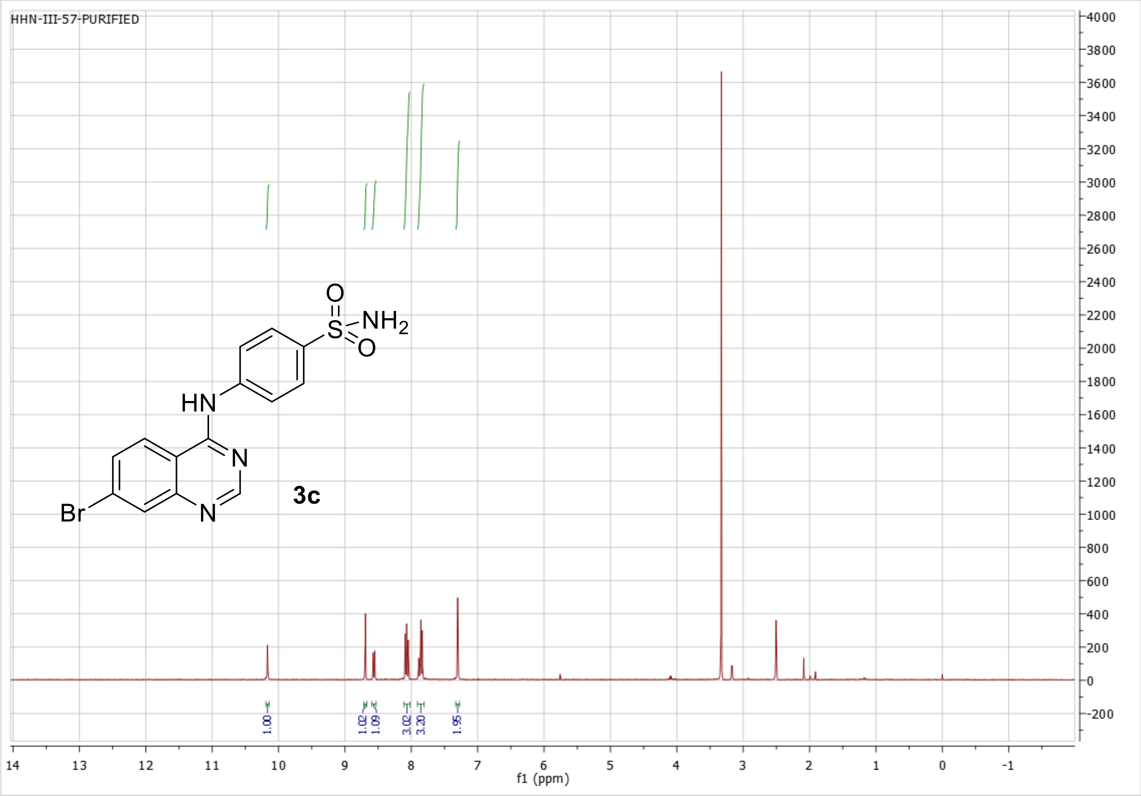
HHN-III-57-PURIFIED
3c
1.00
1.02
1.09
3.02
3.20
1.95
f1 (ppm)

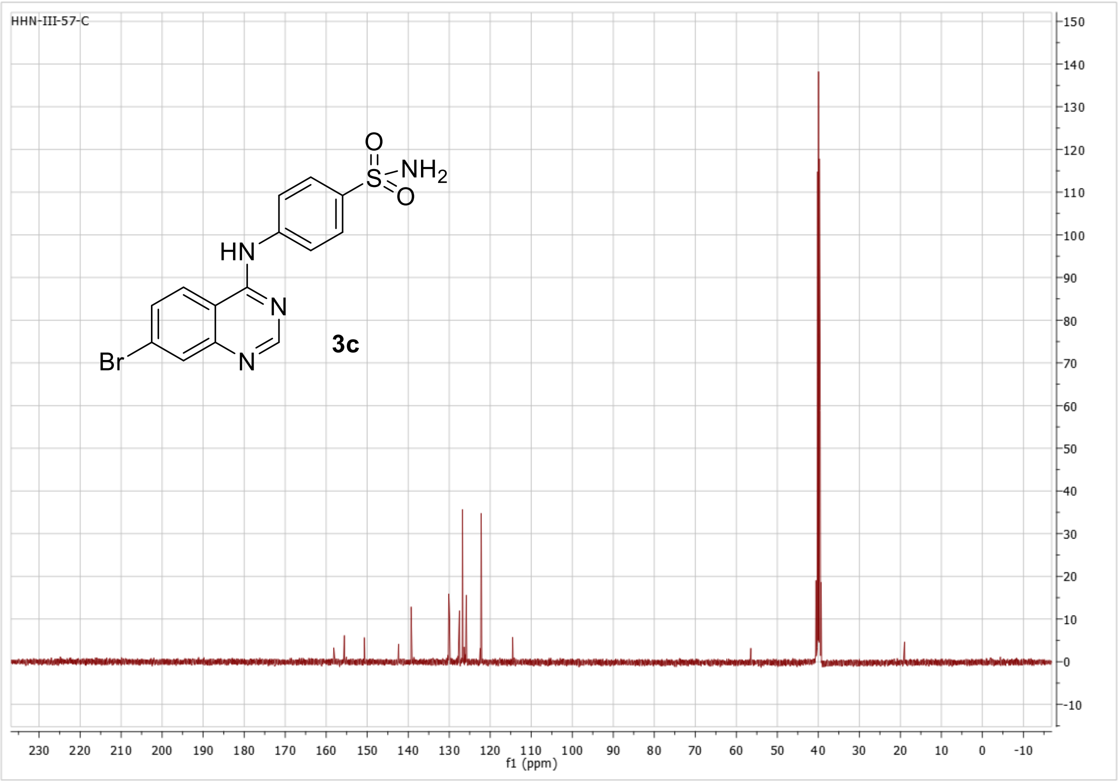
HHN-III-57-C
NH2
HN
N
Br
N
3c
f1 (ppm)

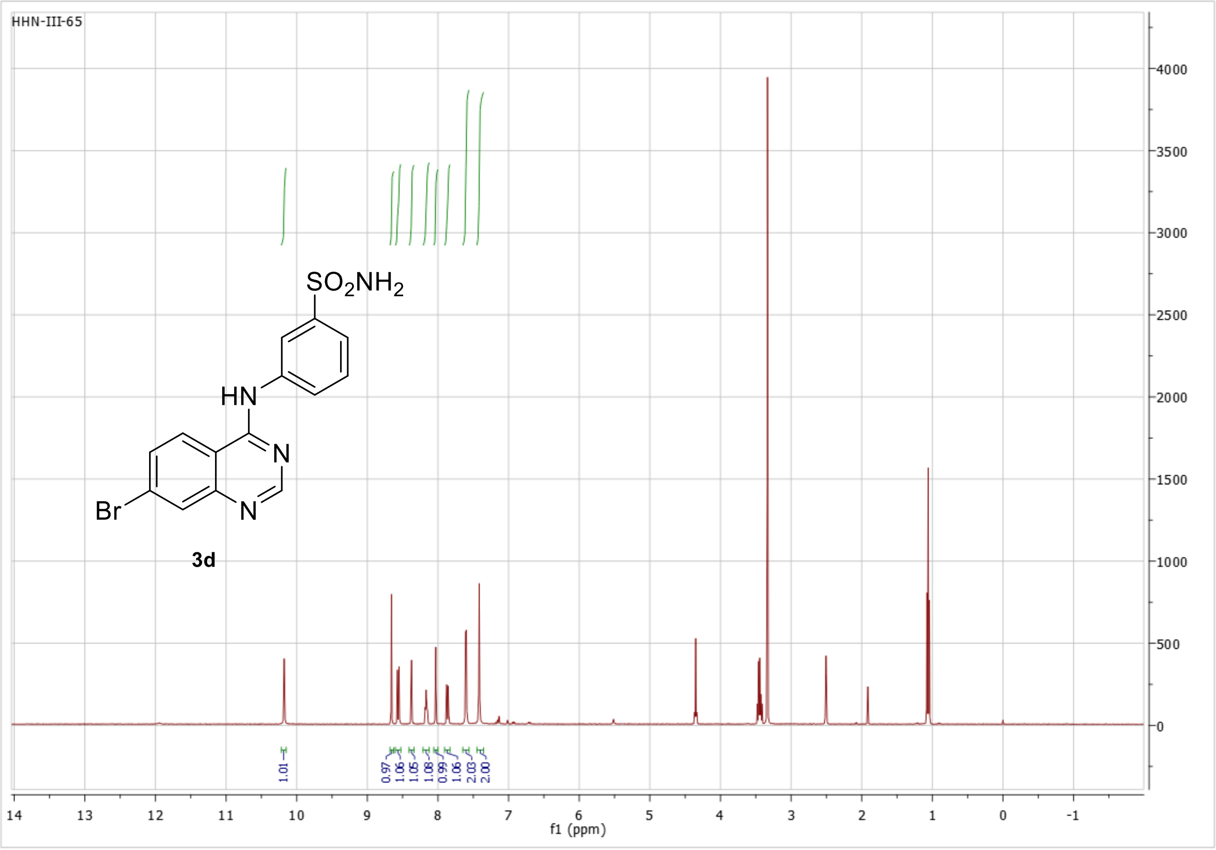
HHN-III-65
SO2NH2
HN
N
Br
N
3d
1.01
0.97
1.06
1.05
1.08
0.99
1.06
2.03
2.00
f1 (ppm)

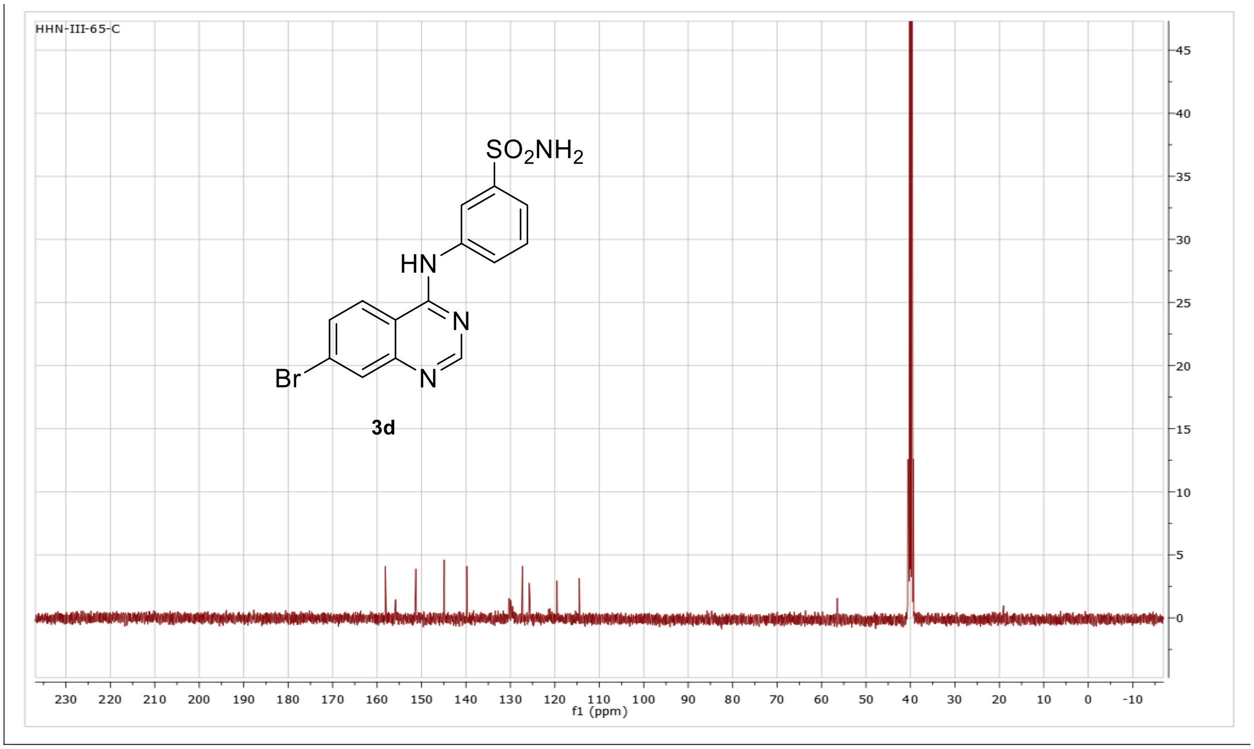
HHN-III-65-C
SO2NH2
HN
N
N
Br
3d
f1 (ppm)

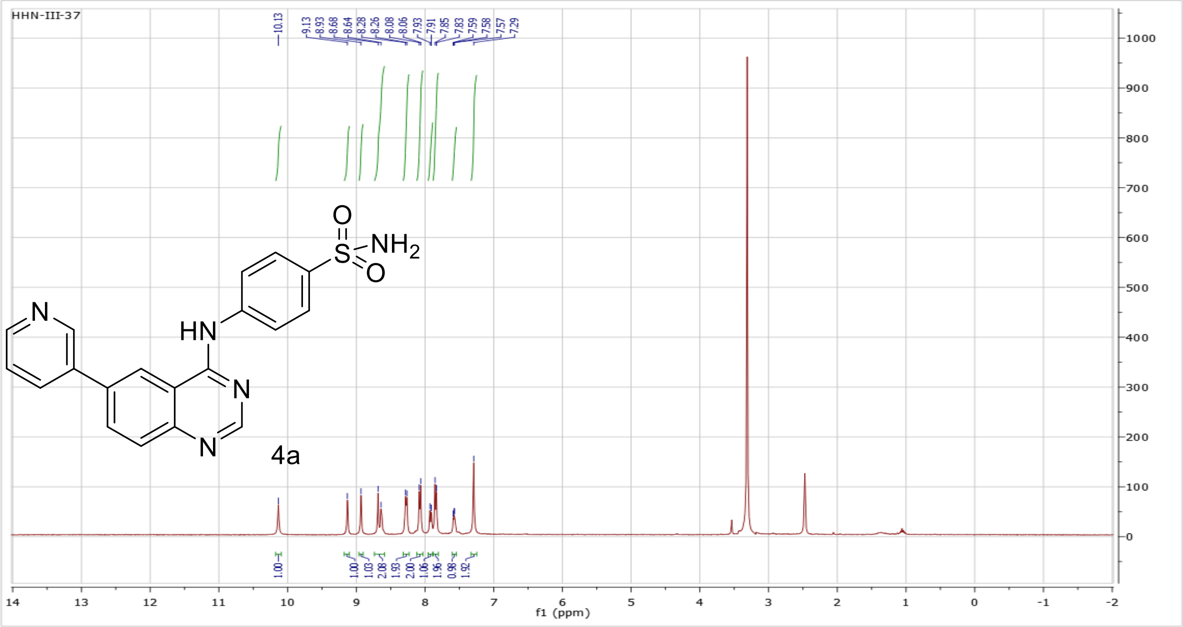
HHN-III-37
10.13
9.13
8.93
8.68
8.64
8.28
8.26
8.08
8.06
7.93
7.91
7.85
7.83
7.59
7.58
7.57
7.29
O
S
NH2
O
N
HN
N
N
4a
1.00
1.00
1.03
2.08
1.93
2.00
1.06
1.96
0.98
1.92
f1 (ppm)

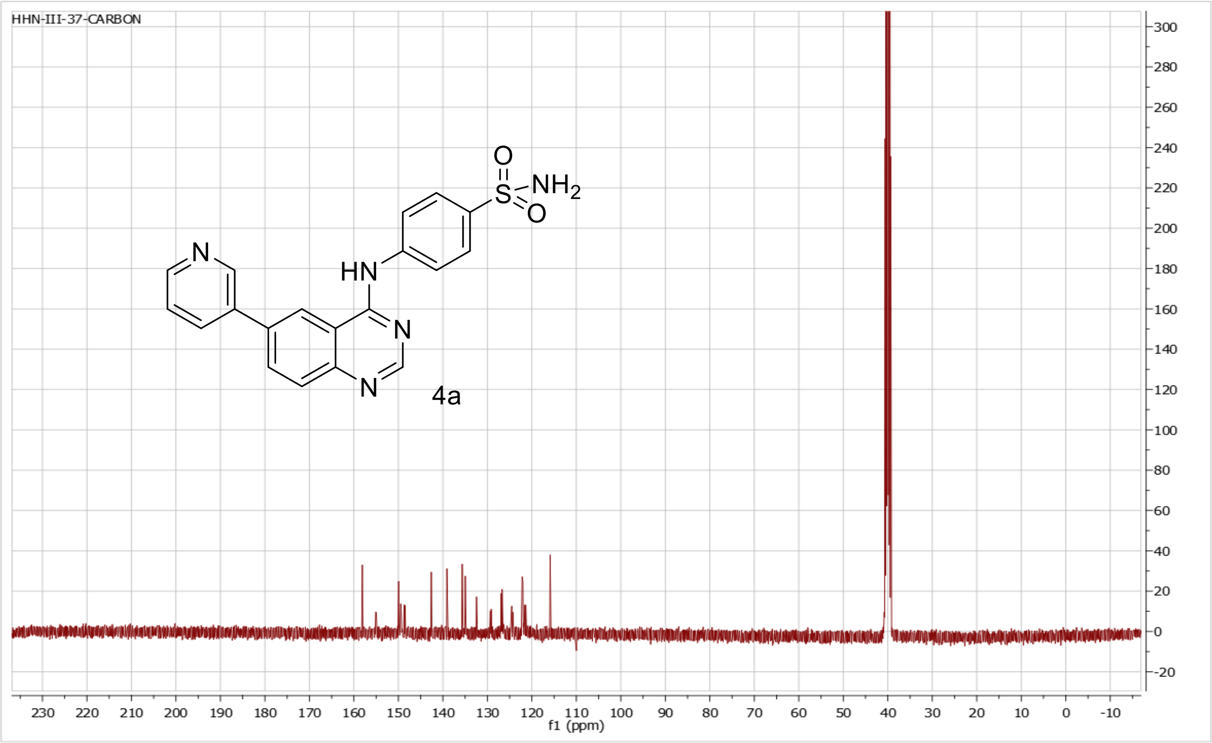
HHN-III-37-CARBON
4a
f1 (ppm)

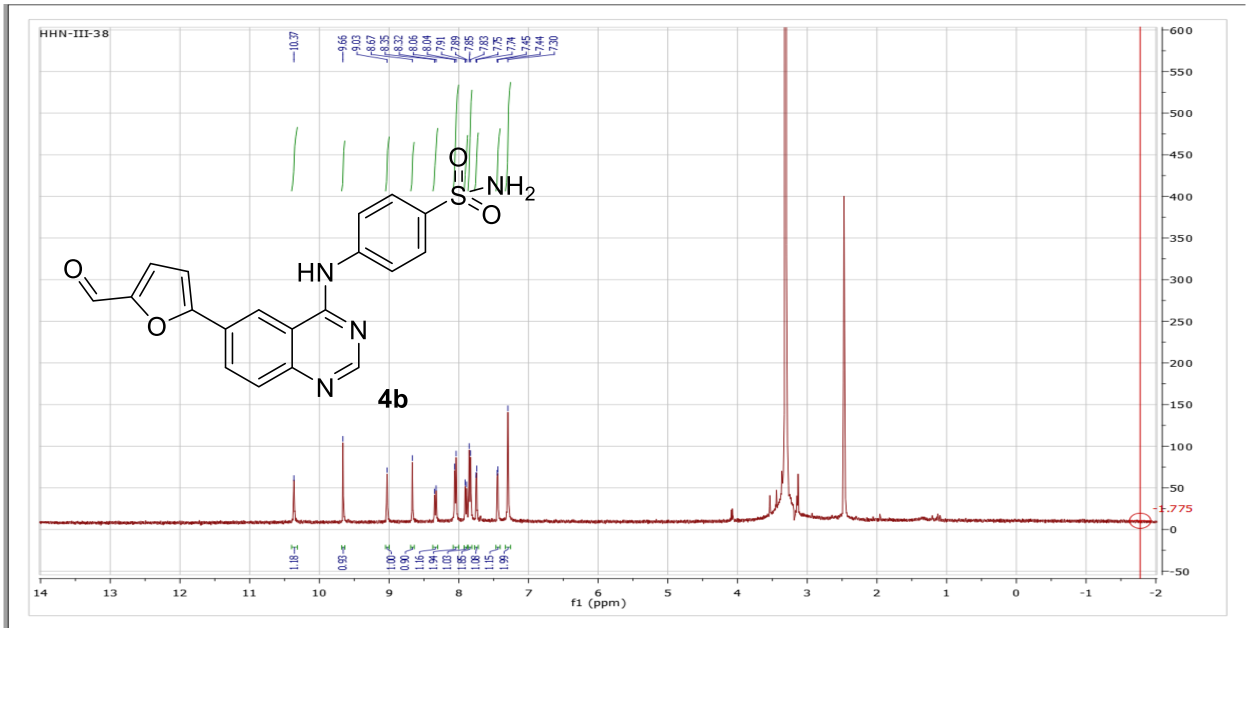
HHN-III-38
10.37
9.66
9.03
8.67
8.35
8.32
8.06
8.04
7.91
7.89
7.85
7.83
7.75
7.74
7.45
7.44
7.30
4b
1.18
0.93
1.00
0.90
1.16
1.94
1.03
1.85
1.08
1.15
1.99
-1.775
f1 (ppm)

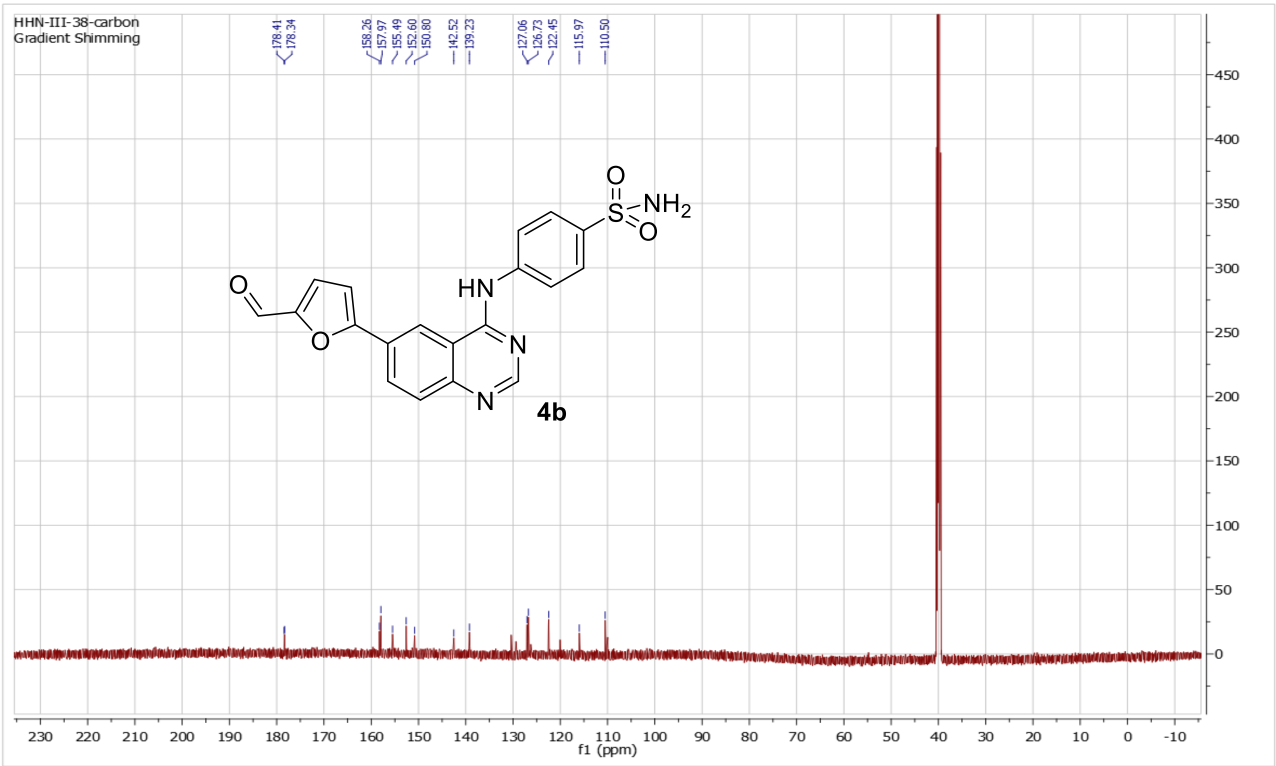
HHN-III-38-carbon
Gradient Shimming
178.41
178.34
158.26
157.97
155.49
152.60
150.80
142.52
139.23
127.06
126.73
122.45
115.97
110.50
4b
f1 (ppm)

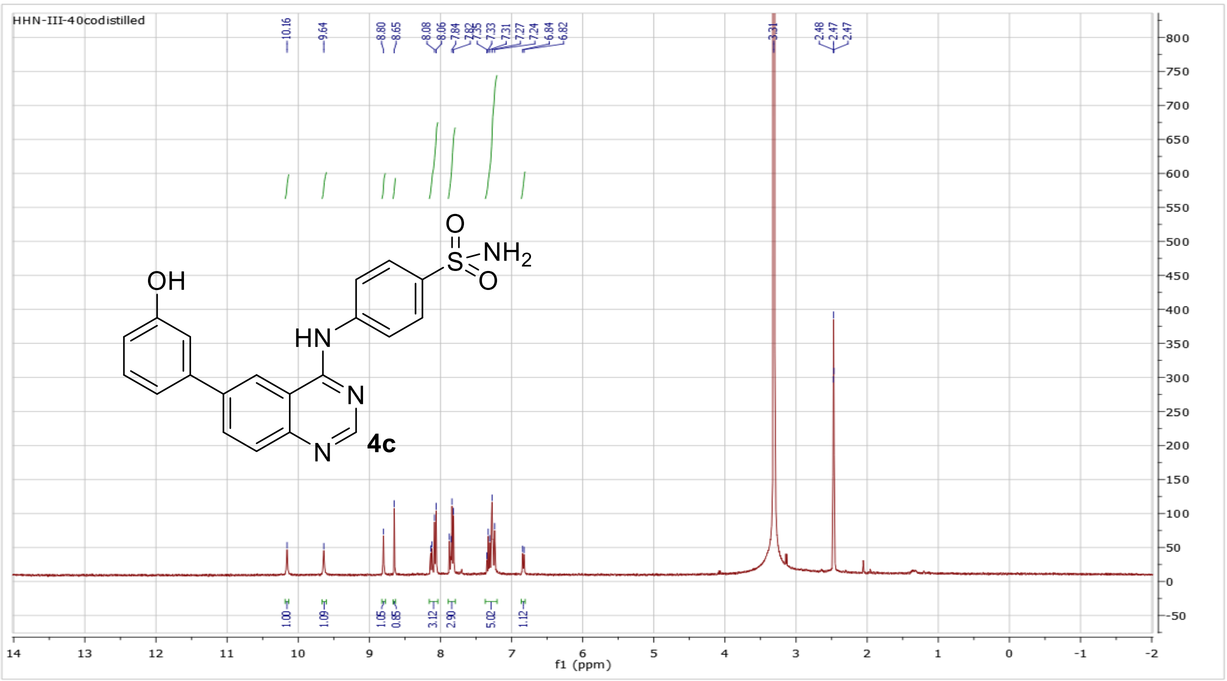
HHN-III-40codistilled
10.16
9.64
8.80
8.65
8.08
8.06
7.84
7.82
7.35
7.33
7.31
7.27
7.24
6.84
6.82
3.31
2.48
2.47
2.47
4c
1.00
1.09
1.05
0.85
3.12
2.90
5.02
1.12
f1 (ppm)

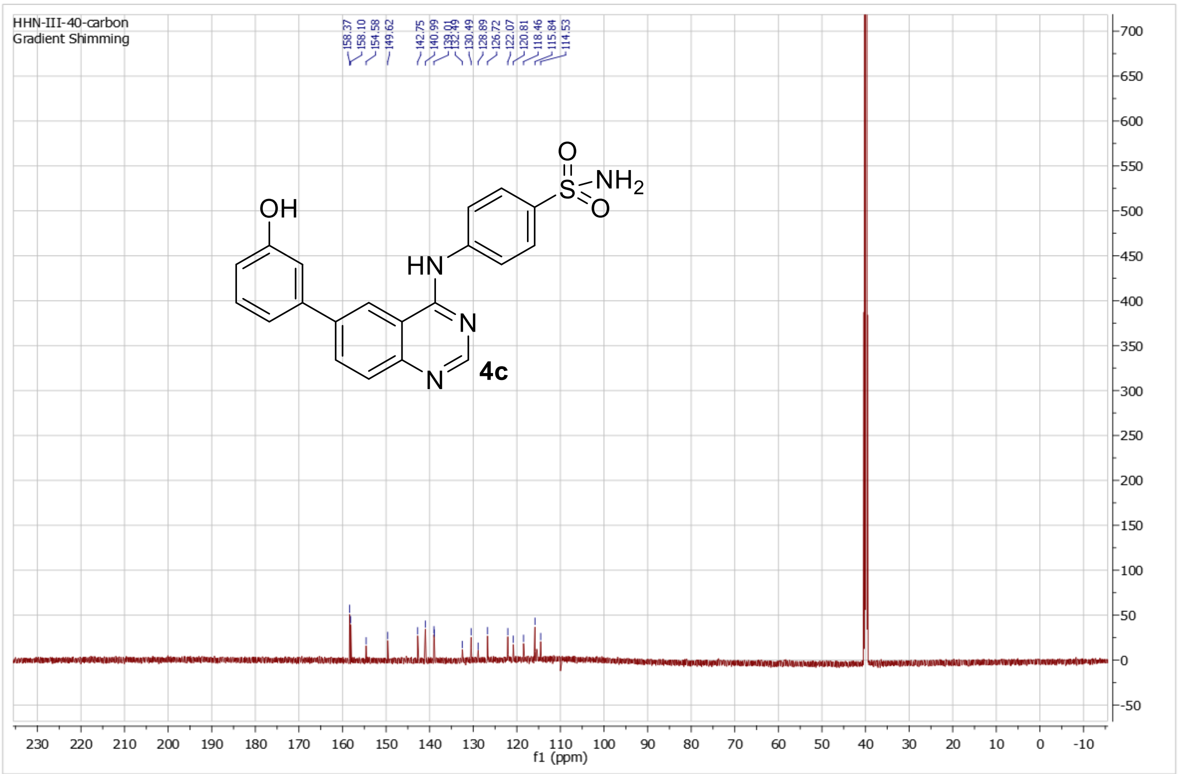

HHN-III-40-carbon
Gradient Shimming
158.37
158.10
154.58
149.62
142.75
140.99
139.01
132.49
130.49
128.89
126.72
122.07
120.81
118.46
115.84
114.53
OH
HN
O
S
NH2
O
N
N
4c
f1 (ppm)

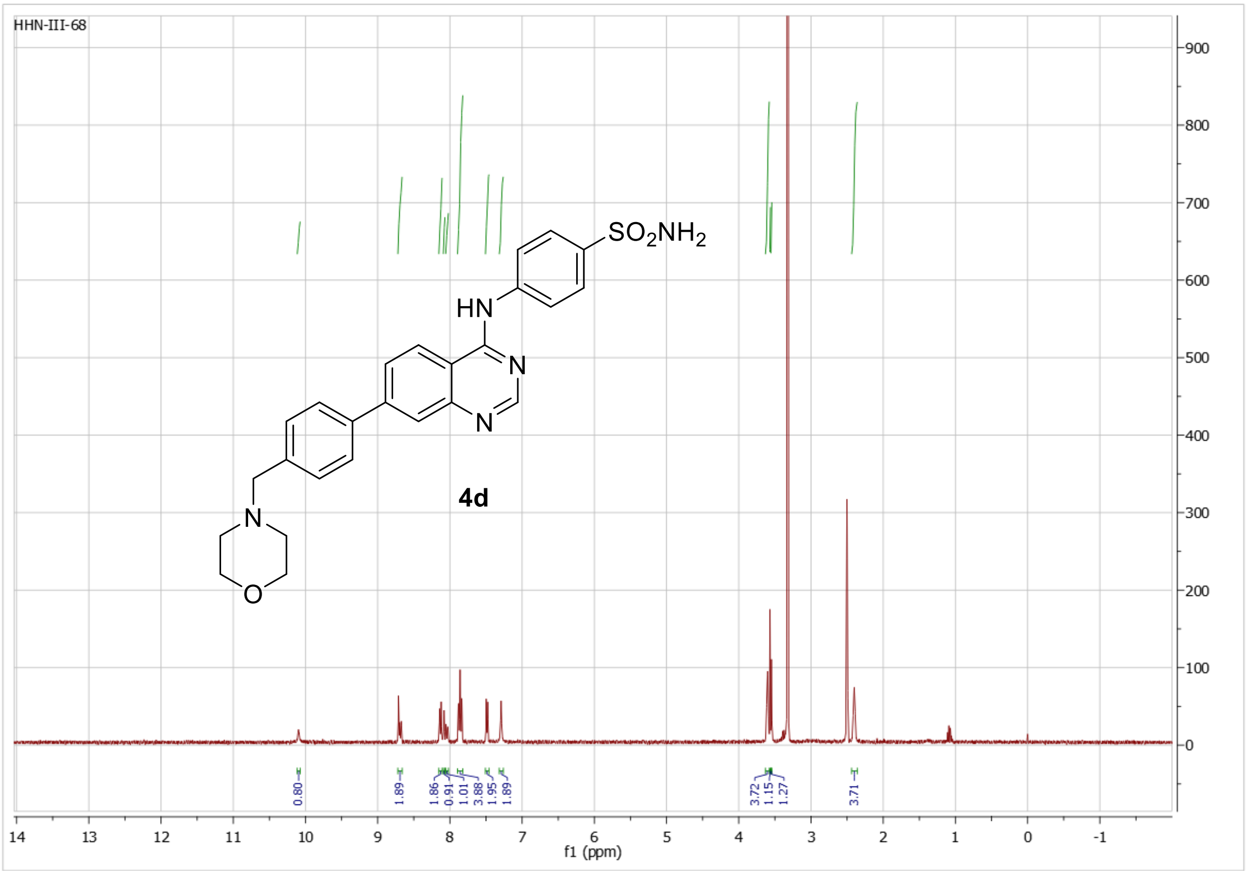

HHN-III-68
SO2NH2
HN
N
N
N
O
4d
0.80
1.89
1.86
0.91
1.01
3.88
1.95
1.89
3.72
1.15
1.27
3.71
f1 (ppm)

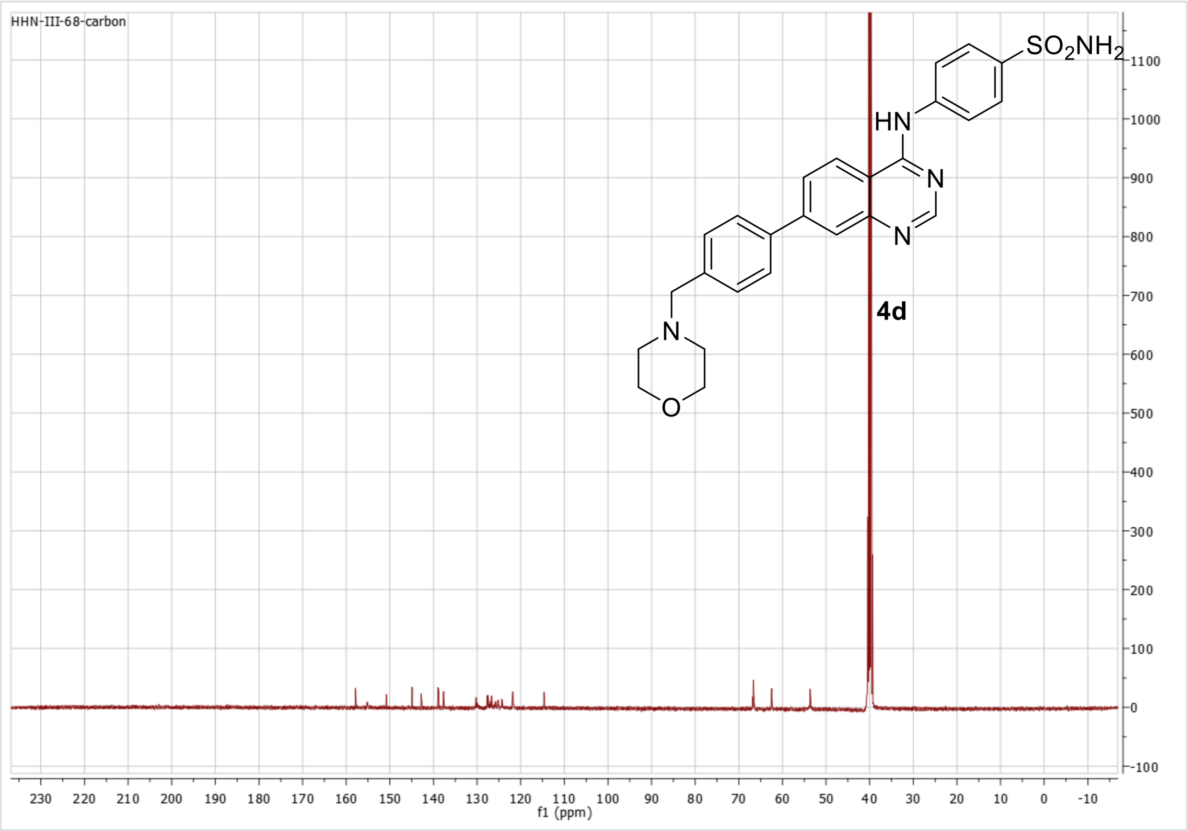
HHN-III-68-carbon
SO2NH2
HN
N
N
N
O
4d
f1 (ppm)

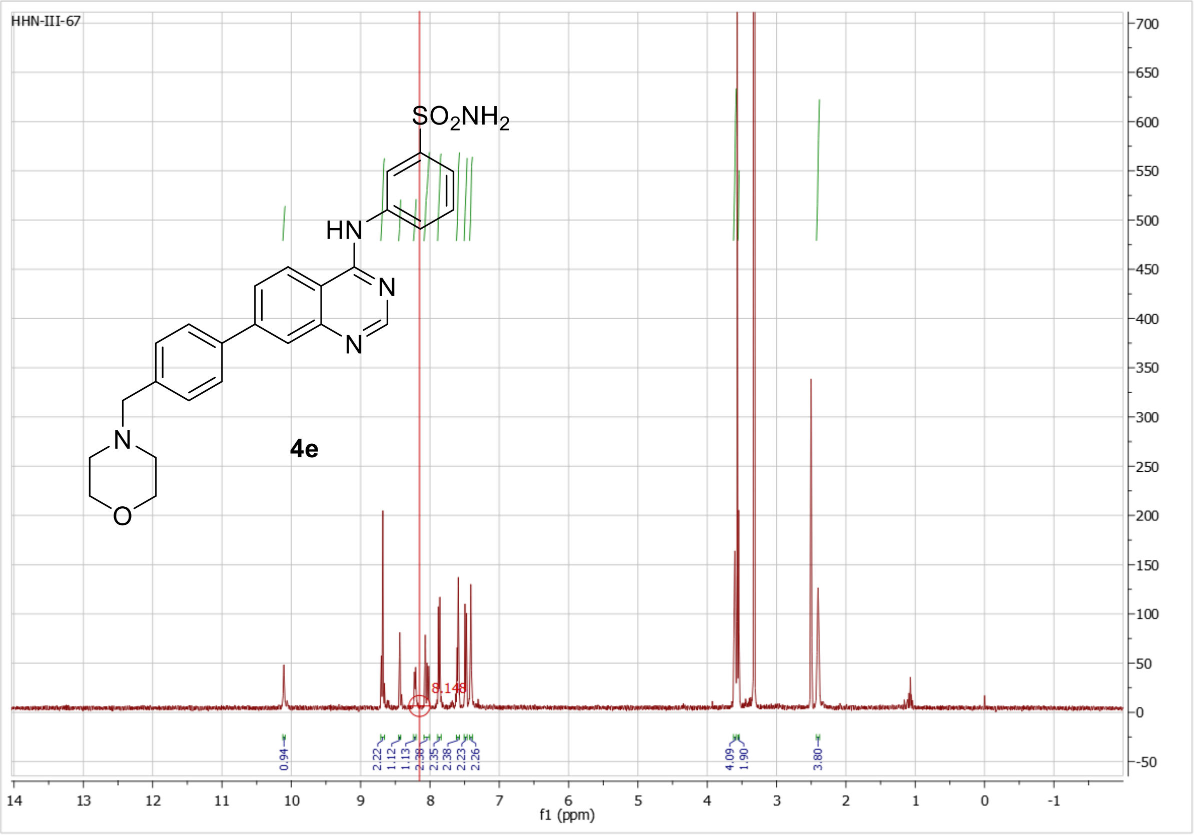
HHN-III-67
SO2NH2
HN
N
N
N
O
4e
8.148
0.94
2.22
1.12
1.13
2.38
2.35
2.38
2.23
2.26
4.09
1.90
3.80
f1 (ppm)

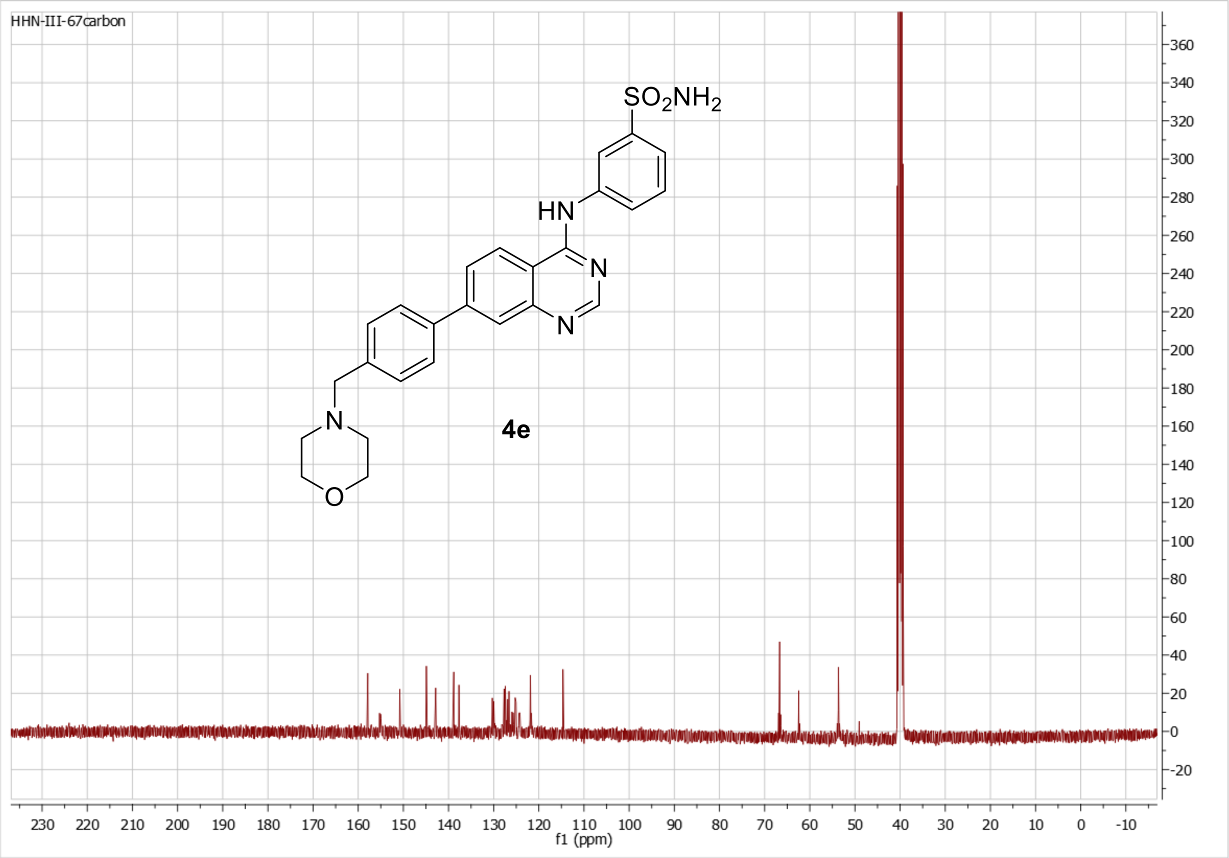

HHN-III-67carbon
SO2NH2
HN
N
N
N
O
4e
f1 (ppm)

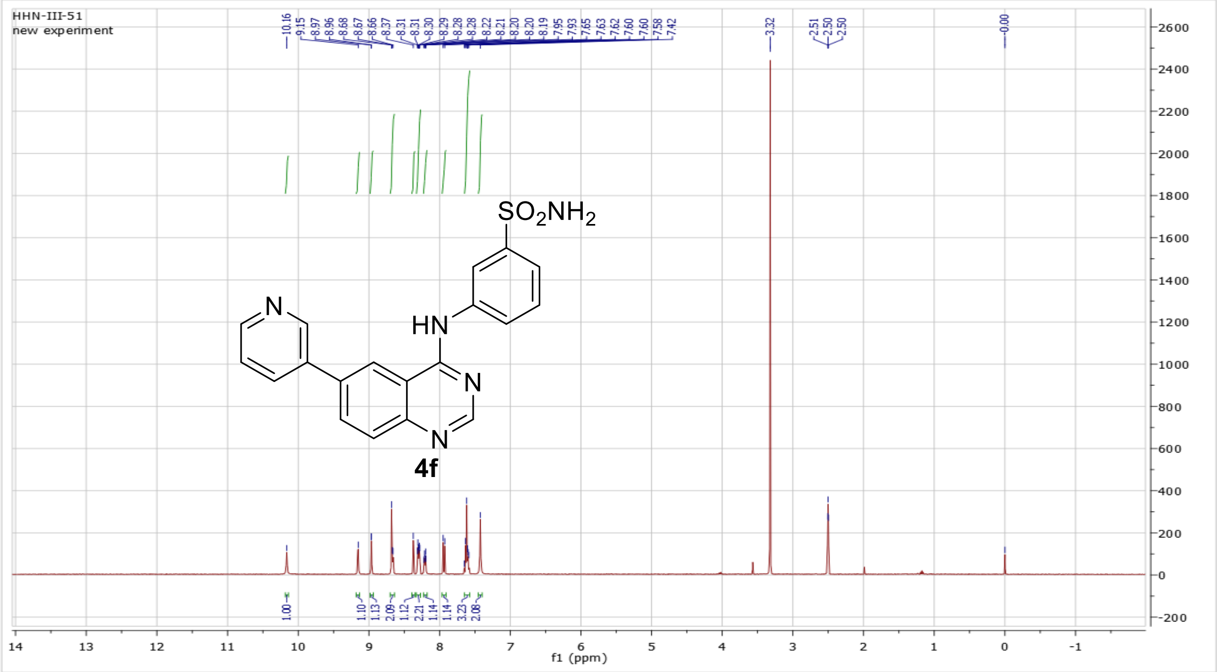

HHN-III-51
new experiment
SO2NH2
HN
N
N
N
4f
f1 (ppm)

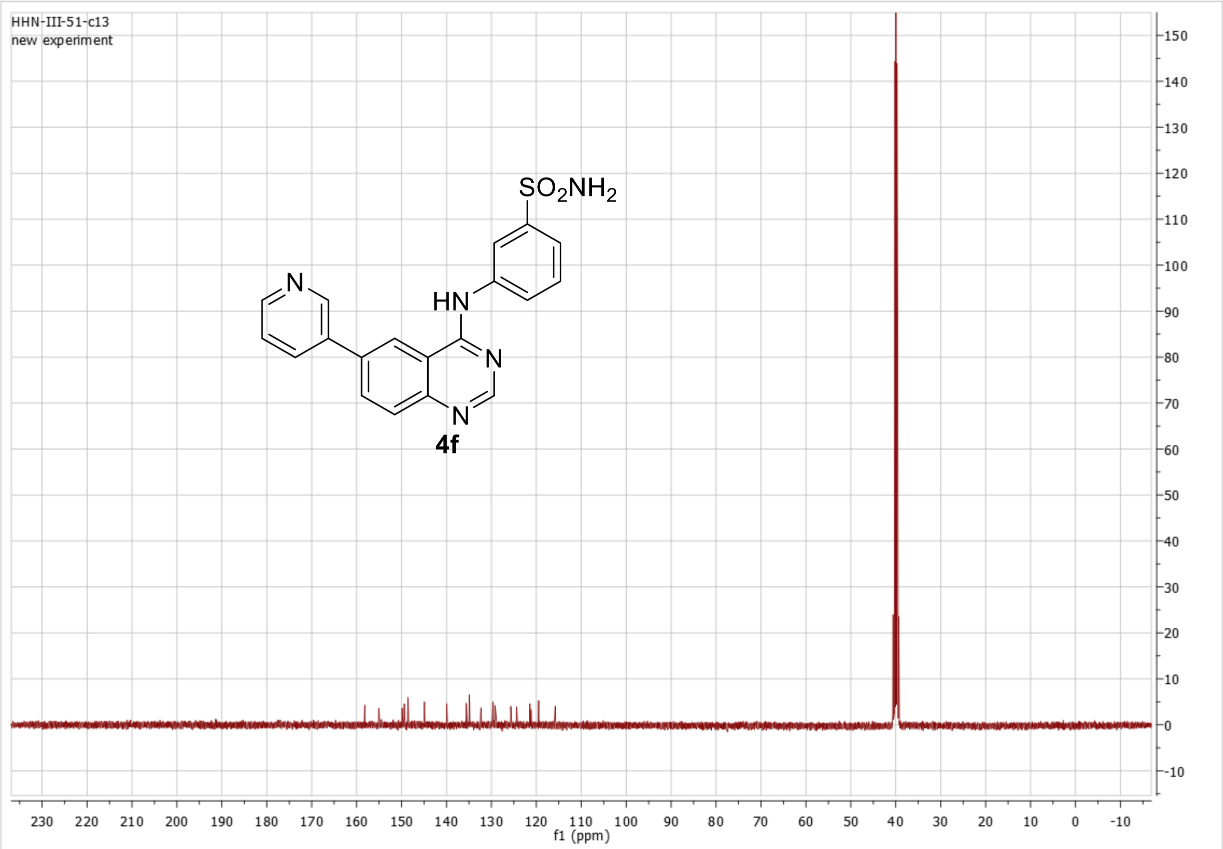
HHN-III-51-c13
new experiment
SO2NH2
HN
N
N
N
4f
f1 (ppm)

3. Representative HPLC purity data of the compounds 3a–d and 4a–f

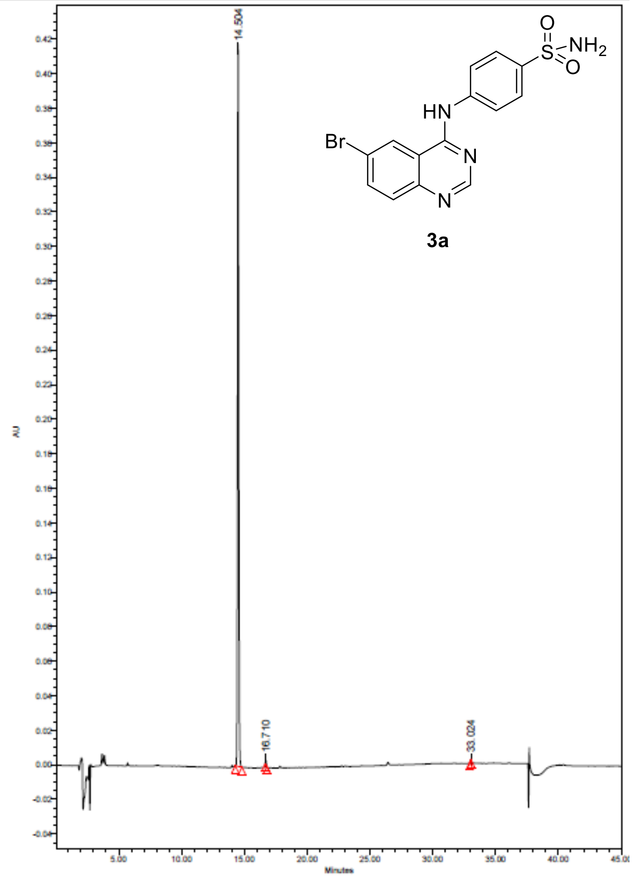

	Name	Retention Time (min)	Purity1 Angle	Purity1 Threshold	PDA Match1 Spect. Name	PDA Match1 Angle	PDA Match1 Threshold	PDA Match1 Lib. Name	Area (µV*sec)	% Area	Height (µV)	Int Type	Amount	Units
1		14.504							2814904	99.46	418996	bb		
2		16.710							11749	0.42	2587	bb		
3		33.024							3664	0.13	851	bb		

	Peak Type	Peak Codes
1	Unknown	
2	Unknown	
3	Unknown	

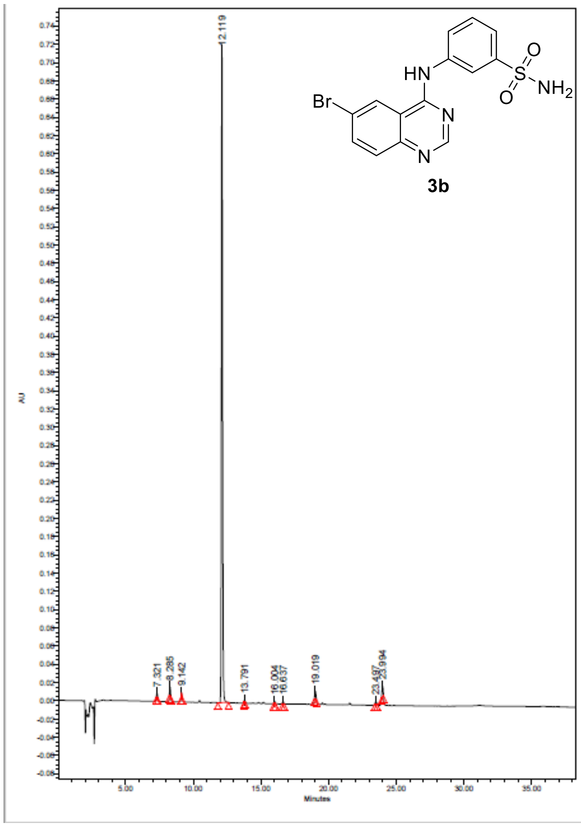

	Name	Retention Time (min)	Purity1 Angle	Purity1 Threshold	PDA Match1 Spect. Name	PDA Match1 Angle	PDA Match1 Threshold	PDA Match1 Lib. Name	Area (µV*sec)	% Area	Height (µV)	Int Type	Amount	Units
1		7.321							19355	0.44	4478	bb		
2		8.285							40947	0.93	9778	bb		
3		9.142							12329	0.28	3927	bb		
4		12.119							4200300	95.11	719106	bb		
5		13.791							4753	0.11	1532	bb		
6		16.004							7963	0.18	1941	bb		
7		16.637							9446	0.21	1950	bb		
8		19.019							46554	1.05	8646	bb		
9		23.497							13240	0.30	2192	bb		
10		23.994							61570	1.39	11239	bb		

	Peak Type	Peak Codes
1	Unknown	
2	Unknown	
3	Unknown	
4	Unknown	
5	Unknown	
6	Unknown	
7	Unknown	

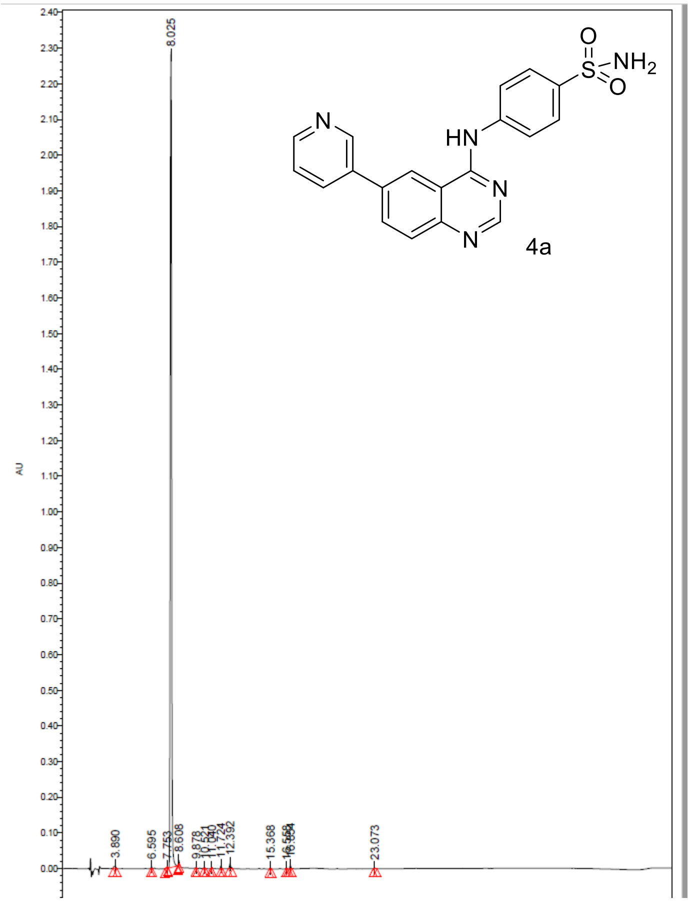

	Name	Retention Time (min)	Purity1 Angle	Purity1 Threshold	PDA Match1 Spect. Name	PDA Match1 Angle	PDA Match1 Threshold	PDA Match1 Lib. Name	Area (µV*sec)	% Area	Height (µV)	Int Type	Amount	Units
1		3.890							63553	0.37	6105	bb		
2		6.595							22251	0.13	4998	bb		
3		7.753							21317	0.12	2894	bb		
4		8.025							16982488	97.84	2329599	bb		
5		8.608							40468	0.23	11424	bb		
6		9.878							4961	0.03	1133	bb		
7		10.521							8423	0.05	1935	bb		
8		11.040							4678	0.03	1354	bb		
9		11.724							34580	0.20	7203	bb		
10		12.392							91000	0.52	13377	bb		
11		15.368							12031	0.07	2334	bb		
12		16.558							8614	0.05	2011	bb		
13		16.854							47861	0.28	9054	bb		
14		23.073							14914	0.09	2018	bb		

	Peak Type	Peak Codes
1	Unknown	
2	Unknown	
3	Unknown	

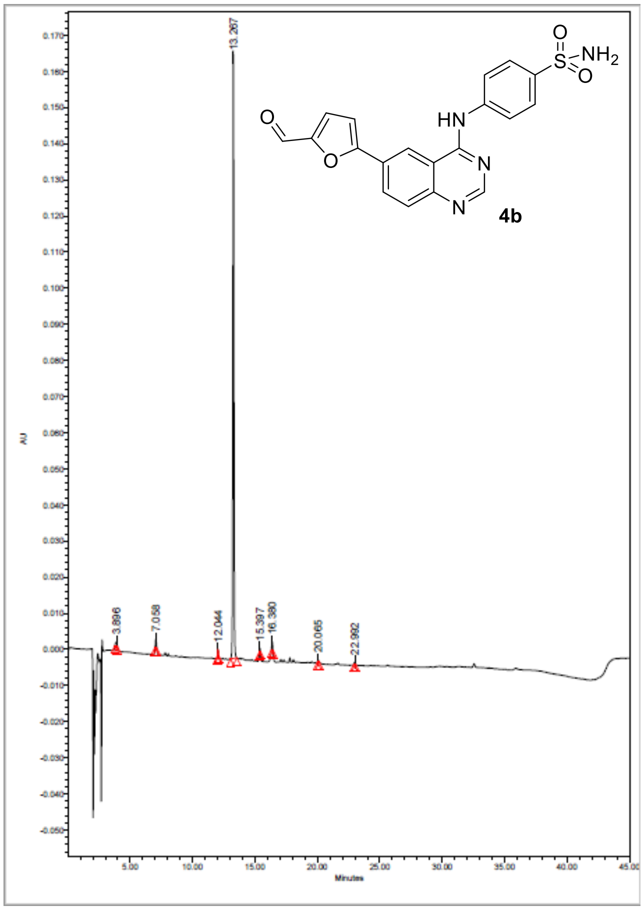

	Name	Retention Time (min)	Purity1 Angle	Purity1 Threshold	PDA Match1 Spect. Name	PDA Match1 Angle	PDA Match1 Threshold	PDA Match1 Lib. Name	Area (µV*sec)	% Area	Height (µV)	Int Type	Amount	Units
1		3.896							9414	0.92	1205	bb		
2		7.058							11571	1.13	2388	bb		
3		12.044							5960	0.58	1692	bb		
4		13.267							977556	95.54	167740	bb		
5		15.397							2373	0.23	950	bb		
6		16.380							11354	1.11	2287	bb		
7		20.065							2506	0.24	490	bb		
8		22.992							2450	0.24	495	bb		

	Peak Type	Peak Codes
1	Unknown	
2	Unknown	
3	Unknown	
4	Unknown	
5	Unknown	
6	Unknown	
7	Unknown	
8	Unknown	

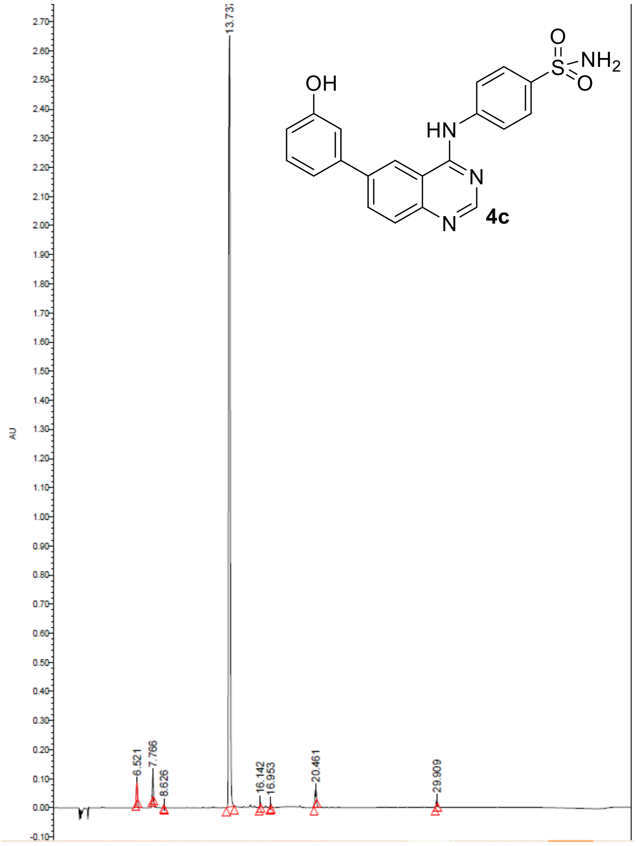

	Name	Retention Time (min)	Purity1 Angle	Purity1 Threshold	PDA Match1 Spect. Name	PDA Match1 Angle	PDA Match1 Threshold	PDA Match1 Lib. Name	Area (µV*sec)	% Area	Height (µV)	Int Type	Amount	Units
1		6.521							356940	1.44	61650	bb		
2		7.766							366231	1.47	78682	bb		
3		8.626							9721	0.04	3802	bb		
4		13.737							23613610	95.10	2674274	bb		
5		16.142							42853	0.17	11096	bb		
6		16.953							30956	0.12	8644	bb		
7		20.461							333272	1.34	43255	bb		
8		29.909							76127	0.31	13868	bb		

	Peak Type	Peak Codes
1	Unknown	
2	Unknown	
3	Unknown	
4	Unknown	
5	Unknown	
6	Unknown	
7	Unknown	
8	Unknown	

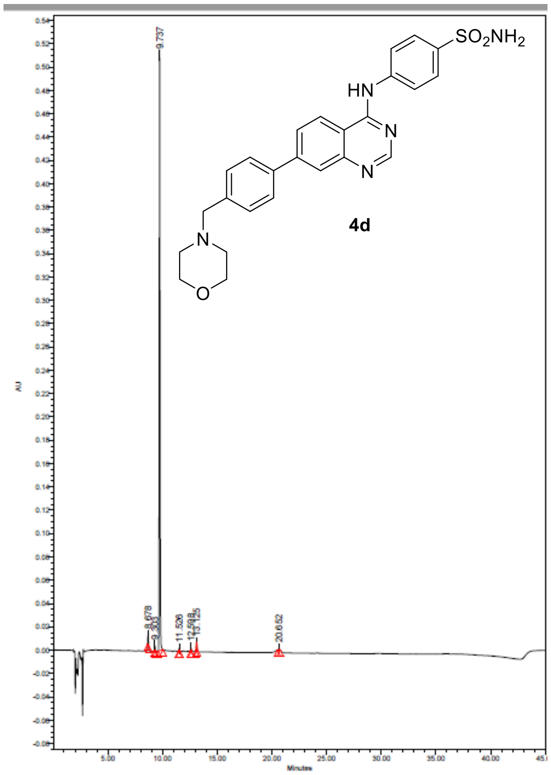

	Name	Retention Time (min)	Purity1 Angle	Purity1 Threshold	PDA Match1 Spect. Name	PDA Match1 Angle	PDA Match1 Threshold	PDA Match1 Lib. Name	Area (µV*sec)	% Area	Height (µV)	Int Type	Amount	Units
1		8.678							28190	1.13	7920	bb		
2		9.303							14303	0.57	3748	bb		
3		9.737							2415863	96.63	506364	bb		
4		11.526							4588	0.18	1490	bb		
5		12.598							7205	0.29	2042	bb		
6		13.125							25605	1.02	6520	bb		
7		20.652							4487	0.18	505	bb		

	Peak Type	Peak Codes
1	Unknown	
2	Unknown	
3	Unknown	
4	Unknown	
5	Unknown	
6	Unknown	
7	Unknown	

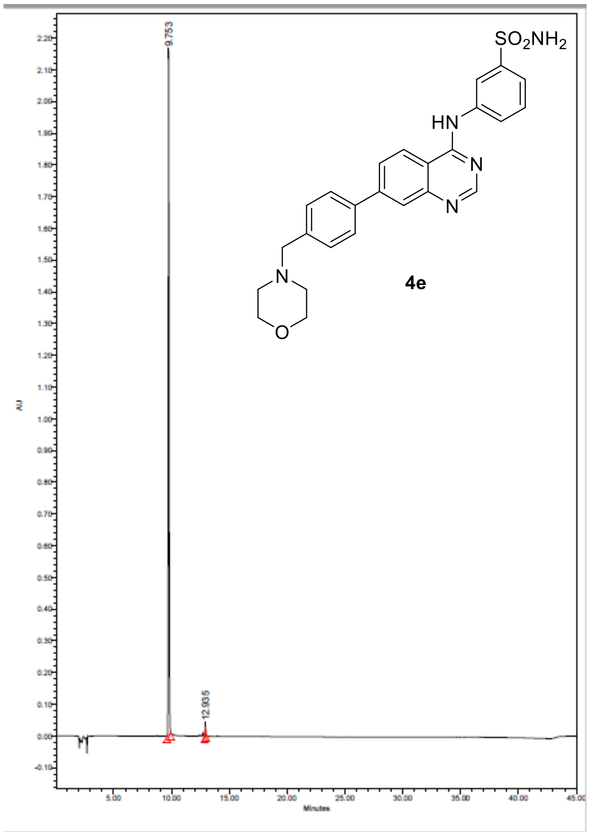

	Name	Retention Time (min)	Purity1 Angle	Purity1 Threshold	PDA Match1 Spect. Name	PDA Match1 Angle	PDA Match1 Threshold	PDA Match1 Lib. Name	Area (µV*sec)	% Area	Height (µV)	Int Type	Amount	Units
1		9.753							11379618	99.15	2167466	bb		
2		12.935							98094	0.85	23466	bb		

	Peak Type	Peak Codes
1	Unknown	
2	Unknown	

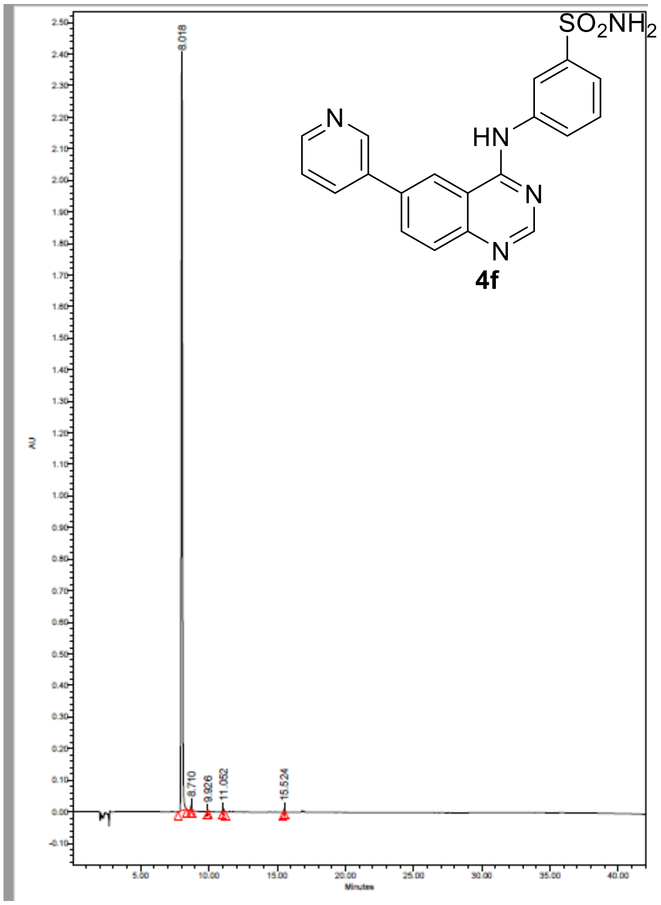

	Name	Retention Time (min)	Purity1 Angle	Purity1 Threshold	PDA Match1 Spect. Name	PDA Match1 Angle	PDA Match1 Threshold	PDA Match1 Lib. Name	Area (μV*sec)	% Area	Height (μV)	Int Type	Amount	Units
1		8.018							14986262	98.93	2414157	bb		
2		8.710							54838	0.36	13790	bb		
3		9.926							6003	0.04	2000	bb		
4		11.052							63323	0.42	9654	bb		
5		15.524							38428	0.25	8270	bb		

	Peak Type	Peak Codes
1	Unknown	
2	Unknown	
3	Unknown	
4	Unknown	
5	Unknown	

4. Representative HRMS data of the compounds 3a–d and 4a–f

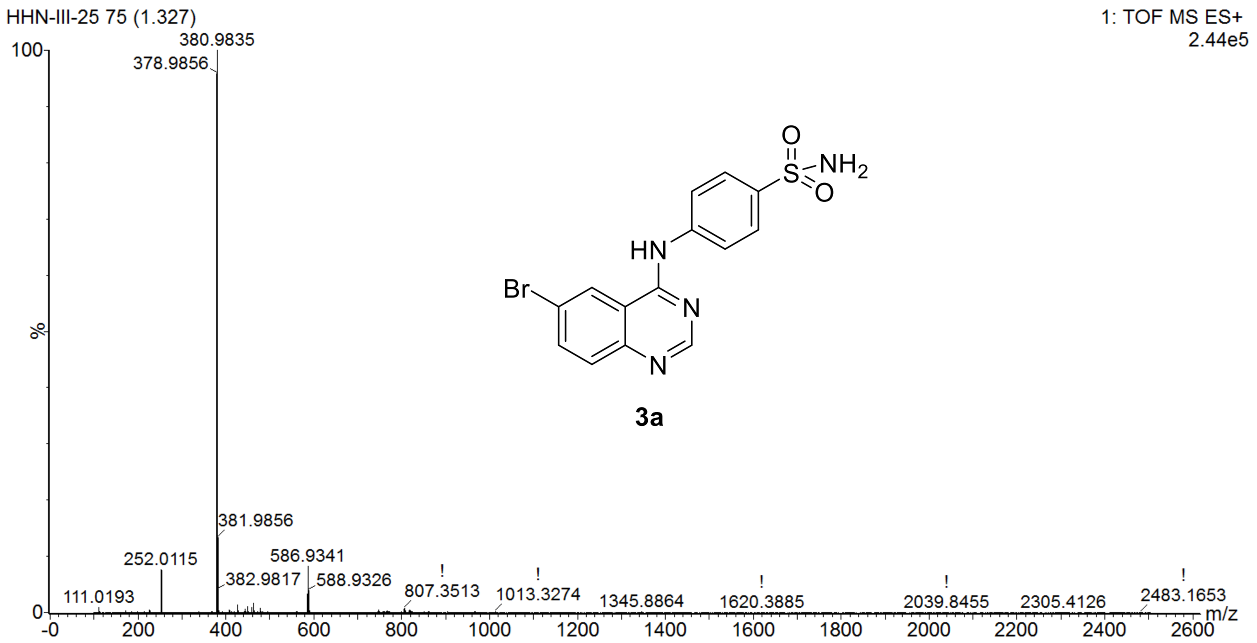

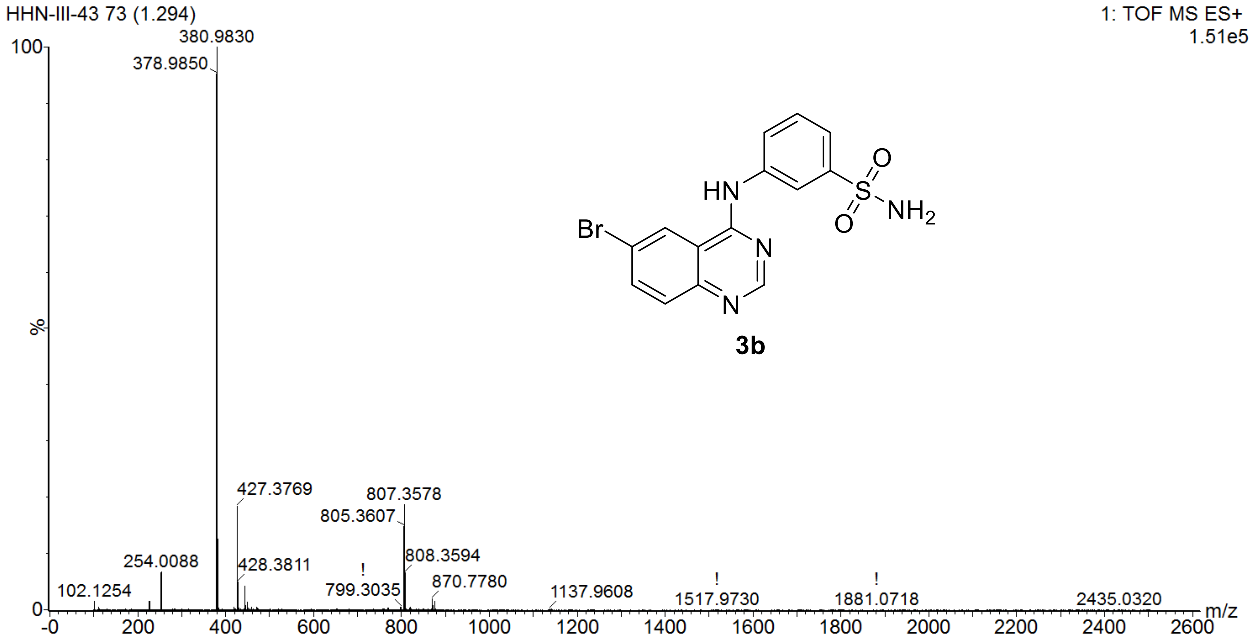

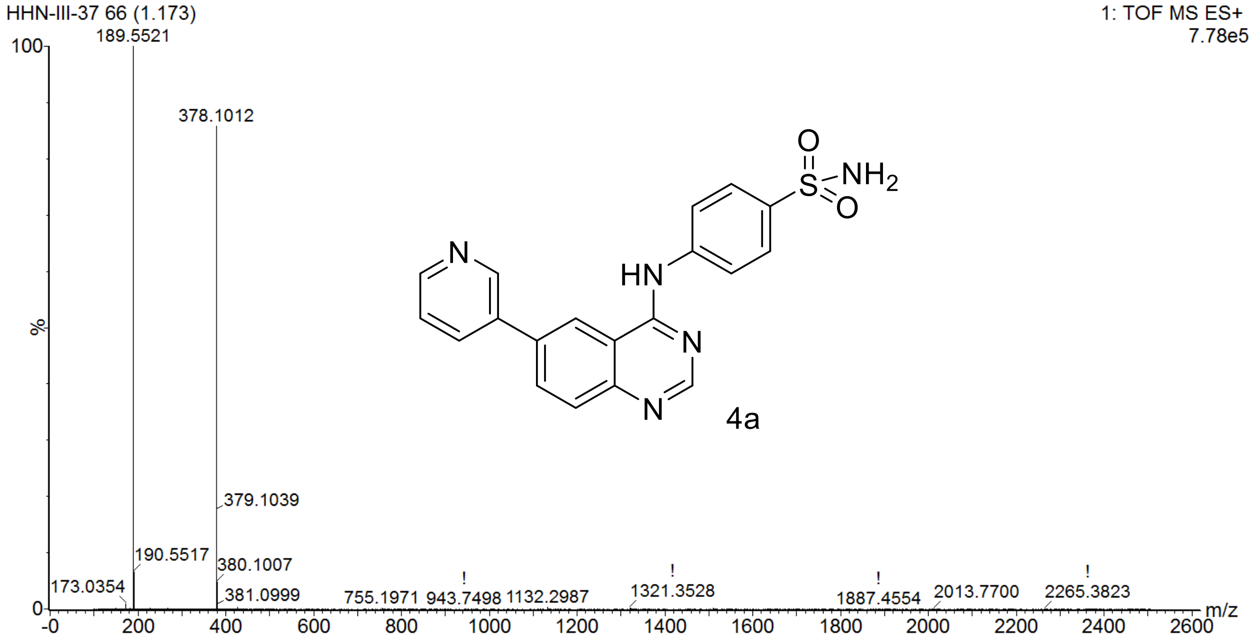
HHN-III-37 66 (1.173)
1: TOF MS ES+
7.78e5
100
%
0
189.5521
378.1012
379.1039
190.5517
380.1007
173.0354
381.0999
755.1971
943.7498
1132.2987
1321.3528
1887.4554
2013.7700
2265.3823
m/z
-0
200
400
600
800
1000
1200
1400
1600
1800
2000
2200
2400
2600
O
S
NH2
O
HN
N
N
N
4a

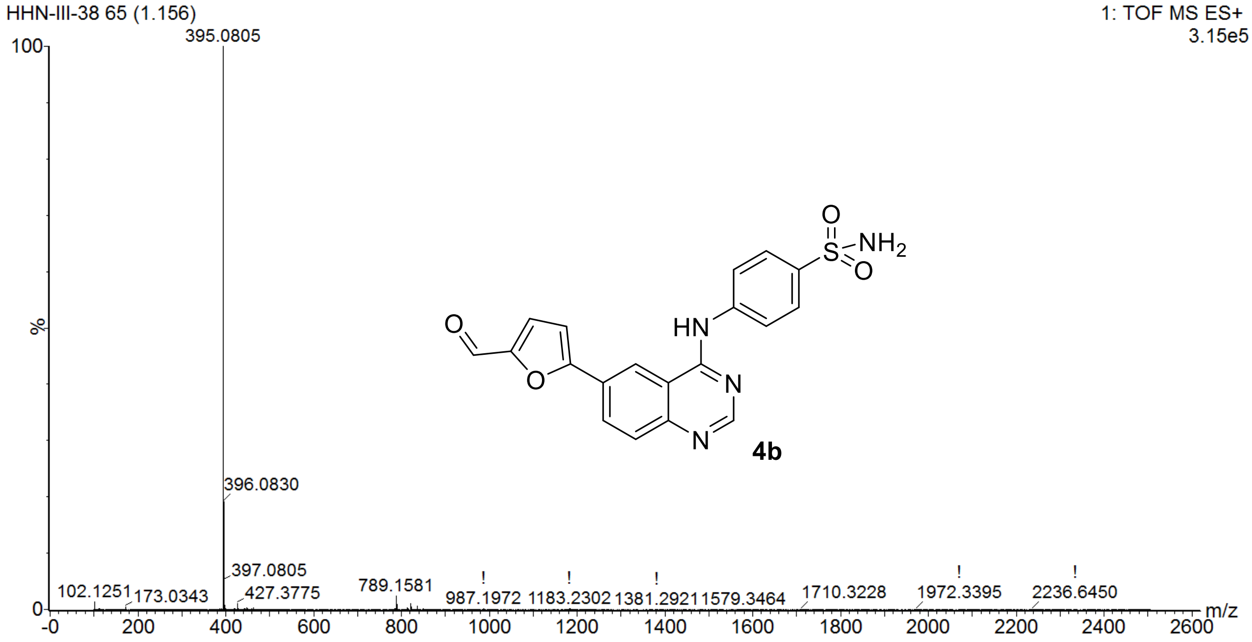
HHN-III-38 65 (1.156)
1: TOF MS ES+
3.15e5
100
%
0
395.0805
396.0830
397.0805
102.1251
173.0343
427.3775
789.1581
987.1972
1183.2302
1381.2921
1579.3464
1710.3228
1972.3395
2236.6450
m/z
-0
200
400
600
800
1000
1200
1400
1600
1800
2000
2200
2400
2600
O
S
NH2
O
HN
O
O
N
N
4b

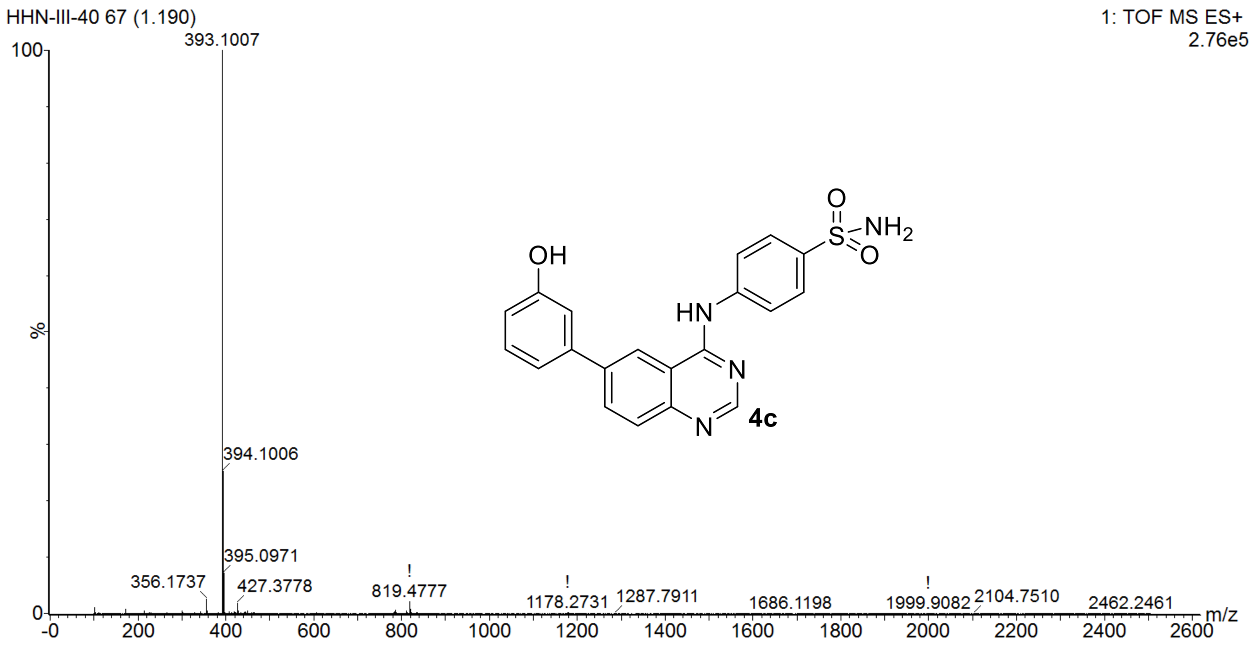
HHN-III-40 67 (1.190)
1: TOF MS ES+
2.76e5
393.1007
394.1006
395.0971
356.1737
427.3778
819.4777
1178.2731
1287.7911
1686.1198
1999.9082
2104.7510
2462.2461
%
m/z
OH
HN
N
N
O
S
NH2
O
4c

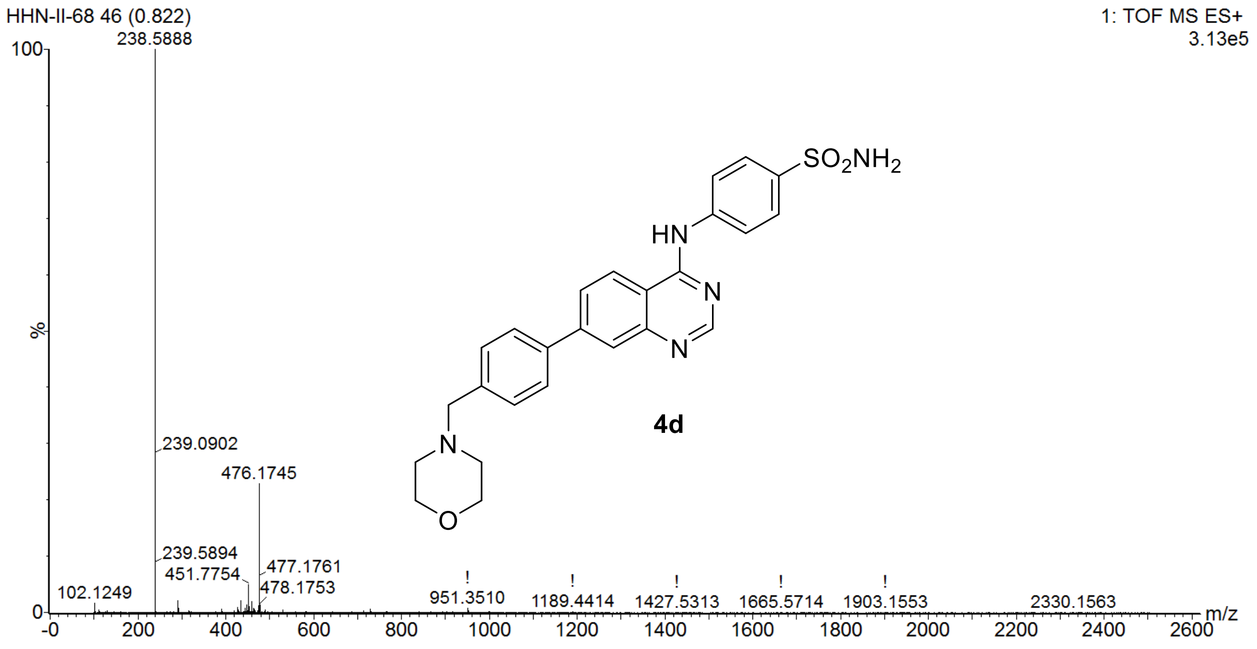
HHN-II-68 46 (0.822)
1: TOF MS ES+
3.13e5
238.5888
239.0902
476.1745
239.5894
451.7754
477.1761
478.1753
102.1249
951.3510
1189.4414
1427.5313
1665.5714
1903.1553
2330.1563
%
m/z
SO2NH2
HN
N
N
N
O
4d

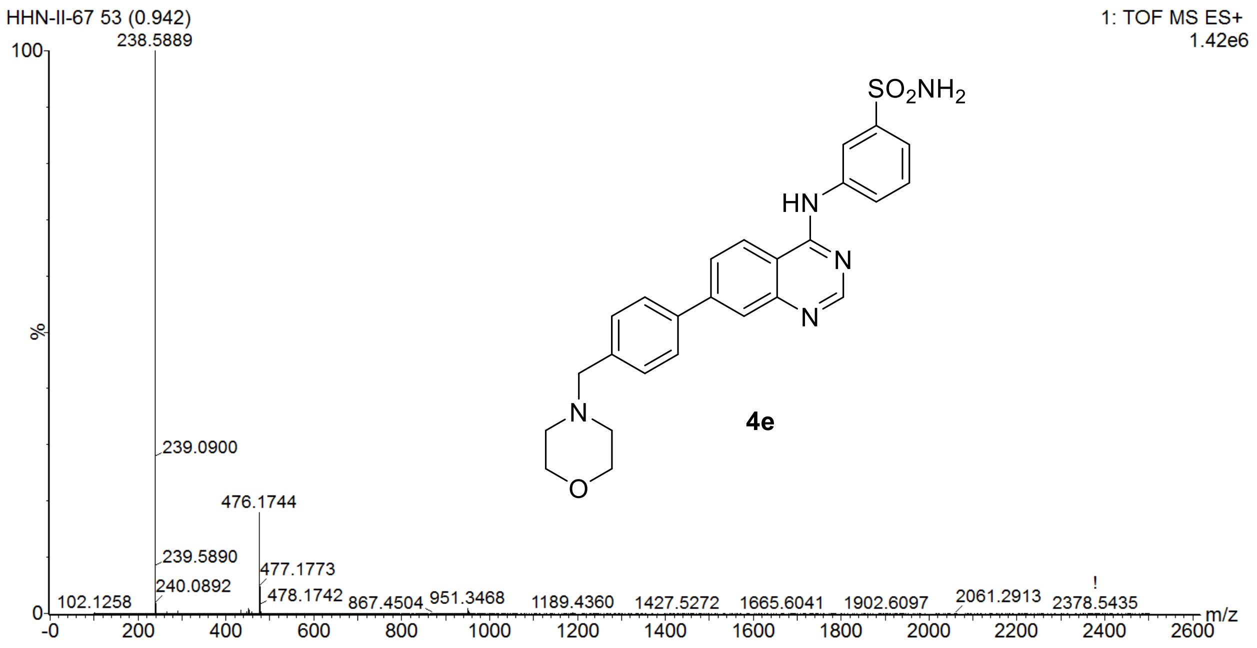
HHN-II-67 53 (0.942)
1: TOF MS ES+
1.42e6
238.5889
239.0900
476.1744
239.5890
477.1773
240.0892
478.1742
102.1258
867.4504
951.3468
1189.4360
1427.5272
1665.6041
1902.6097
2061.2913
2378.5435
%
m/z
SO2NH2
HN
N
N
N
O
4e

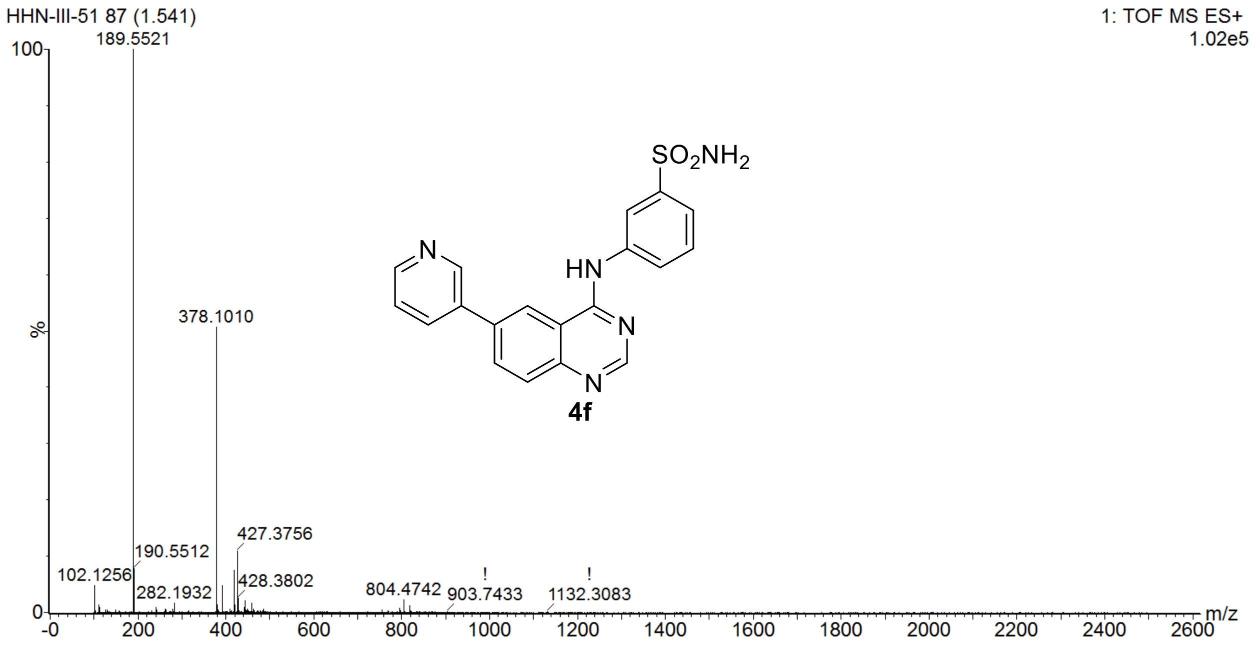
HHN-III-51 87 (1.541)
1: TOF MS ES+
1.02e5
189.5521
378.1010
427.3756
190.5512
428.3802
102.1256
282.1932
804.4742
903.7433
1132.3083
%
m/z
SO2NH2
HN
N
N
N
4f
